# Flagella-mediated secretion of a novel *Vibrio cholerae* cytotoxin affecting both vertebrate and invertebrate hosts

**DOI:** 10.1038/s42003-018-0065-z

**Published:** 2018-06-07

**Authors:** Mitesh Dongre, Bhupender Singh, Kyaw Min Aung, Per Larsson, Regina Miftakhova, Karina Persson, Fatemeh Askarian, Mona Johannessen, Jonas von Hofsten, Jenny L. Persson, Marc Erhardt, Simon Tuck, Bernt Eric Uhlin, Sun Nyunt Wai

**Affiliations:** 1 Department of Molecular Biology and The Laboratory for Molecular Infection Medicine Sweden (MIMS), Umeå University, SE-90187 Umeå, Sweden; 2 Department of Chemistry, Umeå University, SE-901 87 Umeå, Sweden; 3 Faculty of Health Sciences, Department of Medical Biology, Research group of Host-Microbe Interactions, UiT-The Artic University of Norway, 9037 Tromsø, Norway; 4 Umeå Centre for Molecular Medicine (UCMM), Umeå University, SE-90187 Umeå, Sweden; 5 Department of Integrative Medical Biology (IMB), Umeå University, SE-90187 Umeå, Sweden; 6 Helmholtz Centre for Infection Research (HZI), Inhoffenstraße 7, 38124 Braunschweig, Germany

## Abstract

Using *Caenorhabditis elegans* as an infection host model for *Vibrio cholerae* predator interactions, we discovered a bacterial cytotoxin, MakA, whose function as a virulence factor relies on secretion via the flagellum channel in a proton motive force-dependent manner. The MakA protein is expressed from the polycistronic *makDCBA* (*m*otility-*a*ssociated *ki*lling factor) operon. Bacteria expressing *makDCBA* induced dramatic changes in intestinal morphology leading to a defecation defect, starvation and death in *C. elegans*. The Mak proteins also promoted *V. cholerae* colonization of the zebrafish gut causing lethal infection. A structural model of purified MakA at 1.9 Å resolution indicated similarities to members of a superfamily of bacterial toxins with unknown biological roles. Our findings reveal an unrecognized role for *V. cholerae* flagella in cytotoxin export that may contribute both to environmental spread of the bacteria by promoting survival and proliferation in encounters with predators, and to pathophysiological effects during infections.

## Abstract (web summary)

Mitesh Dongre et al. have identified a novel bacterial cytotoxin from *Vibrio chloerae*, whose virulence depends on flagella-mediated secretion. This suggests that the flagella’s role in cytotoxin export could improve survival and proliferation of bacteria when exposed to environmental stress.

## Introduction

V*ibrio cholerae* is an inhabitant of aquatic systems and a causative agent of severe dehydrating diarrhea in humans^1^. *V. cholerae* belonging to the O1 and O139 serogroups expressing cholera toxin (Ctx) and toxin-coregulated pili (Tcp) cause cholera that appears frequently as epidemics^2^. Previously, we discovered that infection with *V. cholerae* O1 El Tor strain C6706 was lethal for the nematode *Caenorhabditis elegans*^3^.

An important virulence factor of many Gram-negative bacteria is the flagellum, a motility organelle that consists of three main parts: the basal body, the flexible hook, and the rigid filament^4^. Closely related to the flagellum is the type-III secretion system, used by several Gram-negative bacteria to deliver effector proteins into eukaryotic host cells^5^. The components of the flagellar-associated type-III secretion system (fT3SS) and the virulence-associated type-III secretion system (vT3SS) are closely related on structural and functional levels^6^. Genes encoding injectisome or vT3SS components have not been found in the genome of several *V. cholerae* O1 strains^7^. A strain lacking the major flagellin subunit FlaA was severely attenuated for colonization in competition assays with wild-type bacteria in an infant mouse infection model^8–10^. However, the putative roles of motility and the flagellar structure *per se* in pathogenicity of *V. cholerae* have remained unclear.

Here, we describe a cytotoxin of *V. cholerae*, MakA (*m*otility *a*ssociated *k*illing factor A) that to our knowledge has not been previously reported. MakA functions as a potent virulence factor and we find it  is pathogenic to *C. elegans* and zebrafish. Our studies demonstrate that the MakA protein is secreted via the fT3SS of the bacterial flagellum thereby being the first described toxin of which we are aware that is exported by this route in *V. cholerae*.

## Results

### Characterization of the *mak* operon in *V. cholerae* quorum sensing-mediated *C. elegans* killing

**Fig. 1:**
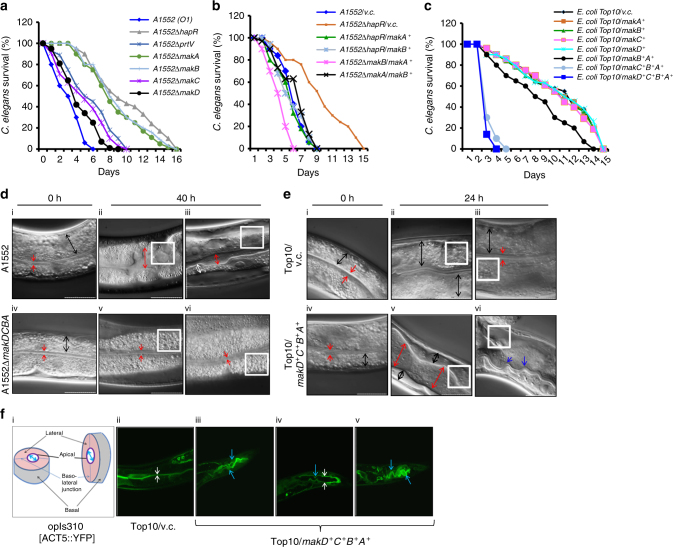
*V. cholerae* and *E. coli* strains harboring the *mak* operon kill *C. elegans* worms. **a** *C. elegans* survival upon feeding on *V. cholerae* wild-type O1 strain A1552 and A1552 containing deletions of indicated genes. **b** *C. elegans* survival upon feeding on different A1552 mutants expressing different *mak* genes; v.c. stands for vector control. **c** *C. elegans* survival upon feeding on *E. coli* Top10 strains harboring different *mak* genes. Data represent average survival percentage of three separate experiments. **d**, **e** Nomarski micrographs of *C. elegans* worms treated with *V. cholerae* A1552 (D, upper panels), A1552∆*makDCBA* (D, lower panels), *E. coli* Top10/vector (E, upper panels), *E. coli* Top10/*makD*^*+*^*C*^*+*^*B*^*+*^*A*^*+*^ (E, lower panels); 0 h after the treatment (D and E, i and iv), 40 h after the treatment (D; ii, iii, v, vi). 24 h after the treatment (Fig. E; ii, iii, v, vi). **f**. Fluorescence micrographs (ii, iii, iv, v) of *opIs310* [*act-5*::YFP] worms fed for 24 h with *E. coli* Top10/vector (ii) or Top10/*makD*^*+*^*C*^*+*^*B*^*+*^*A*^*+*^ (iii, iv, v). White boxes; lipid droplets. Red arrows; the boundaries of the intestinal lumen. Black arrows; the breadth of the intestinal cell. Blue arrows; convolution of intestinal lumen

We reported previously that killing of *C. elegans* by *V. cholerae* O1 El Tor strains was mediated by a quorum sensing regulator, HapR, through its downstream effector PrtV^3^. The A1552∆*hapR* derivative was strongly attenuated when compared to the wild-type strain A1552 whereas the A1552∆*prtV* mutant strain appeared only moderately attenuated (Fig. 1a). This suggested that some additional HapR-regulated factor(s) causing *C. elegans* killing may be expressed by the wild-type *V. cholerae* strain A1552 .

*V. cholerae* contains several putative HapR-regulated genes^11^. Analyses of HapR targets indicated that the most strongly induced target locus was *vca0880*, which is found in an operon with *vca0881*, *vca0882*, and *vca0883* of unknown biological function^12^. We investigated the potential role of this putative polycistronic operon, here denoted as the *makDCBA* operon, including four *mak* (*m*otility-*a*ssociated *k*illing factor) genes *vca0880* (*makD*), *vca0881* (*makC*), *vca0882* (*makB*), and *vca0883* (*makA*). Firstly, we tested the level of MakA in the Δ*hapR* mutant derivative by immunoblot analysis. MakA expression was dramatically reduced implying that *makA* was indeed regulated by HapR (Supplementary Fig. 1A lane 2). To investigate the biological significance of the *makDCBA* locus, we generated in-frame deletion mutants. The ∆*makB* and ∆*makA* single mutants showed a clear attenuation comparable with that of the *A1552ΔhapR* mutant whereas the effect of ∆*makD* or ∆*makC* was minimal (Fig. 1a). The ∆*makDC* mutant was slightly more impaired for *C. elegans* killing (Supplementary Fig. 1B) than either ∆*makD* or ∆*makC* alone (Fig. 1a). Moreover, ∆*makCB*, ∆*makDCB*, ∆*makBA*, and ∆*makDCBA* mutants were clearly attenuated (Supplementary Fig. 1B).

Our bioinformatic analyses of MakA, MakB, MakC, and MakD indicated that these proteins are conserved in Vibrio species. To ascertain a role of the *makDCBA* operon, we investigated whether the *makD*^*+*^*, makC*^*+*^*, makB*^*+*^, *and makA*^*+*^ genes could restore the ability of the A1552∆*hapR* mutant to kill *C. elegans*. Constitutive expression of *makA*^*+*^ or *makB*^*+*^ completely restored the ability of the A1552∆*hapR* mutant to kill worms (Fig. 1b), but constitutive expression of *makC*^*+*^ or *makD*^*+*^ failed to do so (Supplementary Fig. 1C).

Next, we reciprocally complemented *V. cholerae* A1552Δ*makA* and A1552Δ*makB* with the *makB+* and *makA+* clones, respectively. Expression of MakA in A1552Δ*makB* restored *C. elegans* killing more efficiently than MakB did in the A1552Δ*makA* mutant (Fig. 1b), indicating that MakA has a more toxic effect in *C. elegans* than MakB.

The attenuated *C. elegans* killing observed for A1552∆*makA* and A1552∆*makB* mutants was phenotypically indistinguishable from that of A1552Δ*hapR*, suggesting that there is a dominant contribution of the *makDCBA* operon to HapR-mediated nematode killing. Postulating that MakA might be secreted by a general mechanism, we expressed MakD, MakC, MakB and MakA in *E. coli* Top10 and examined *C. elegans* killing potential. *E. coli* expressing individual *makD*, *makC*, *makB*, or *makA* genes were non-toxic, and simultaneous expression of *makB* and *makA* failed to kill *C. elegans* (Fig. 1c). However, tandemly cloned *makCBA* or *makDCBA* loci exhibited strong toxicity (Fig. 1c). These results, together with those obtained with *V. cholerae* mutants (Supplementary Fig. 1B and Fig. 1a), indicate that MakD or MakC is required with MakA and MakB for efficient killing of *C. elegans*.

### The effect of *mak* operon proteins on *C. elegans* intestinal tissue

To understand how expression of the *makDCBA* genes kill *C. elegans*, we investigated morphological changes that occur over time. In worms treated with *V. cholerae* A1552, after 40 h, the intestinal lumen was expanded and filled with bacteria and liquid (Fig. 1d, ii and iii). No changes were seen in *C. elegans* treated with the Δ*makDCBA* mutant (Fig. 1d, v and vi). In worms treated with *E. coli* the dilatation of the intestinal lumen was more pronounced and occured more quickly (within 24 h) (Fig. 1e, v and vi). Worms fed with *E. coli* habouring the vector control were normal in appearance (Fig. 1e, ii and iii). In worms fed with bacteria expressing all four Mak proteins, we also consistently observed several other defects. In particular, the lipid droplets visible normally in the cytoplasm of intestinal cells were greatly reduced in worms feeding on the MakA-expressing bacterial strains (compare areas highlighted with white frames in Fig. 1d: ii and iii vs. v and vi; and in Fig. 1e: ii and iii vs. v and vi). If the lumen was not extended, the apical membrane of the lumen was greatly expanded and it was invariably highly convoluted especially in the worms treated with *E. coli* over-expressing Mak proteins (Fig. 1e, vi, blue arrows). The intestinal cytoplasm was also notably thinner: the basolateral surfaces were appreciably further away from the hypodermal cells underlying the cuticle (Fig. 1e, v and vi). A fluorescent marker for cell junctions, AJM-1::GFP, showed that when the worms were fed with *E. coli* over-expressing all four Mak proteins, although the intestine was grossly abnormal, the tight junctions between neighboring intestinal cells were not disrupted (Supplementary Fig. 1D). To test if the apical surface of the intestine was affected, we used a fluorescent marker, the ACT-5::YFP protein. The convoluted intestinal apical surface was clearly seen in the worms fed with *E. coli* habouring the *mak* operon (Fig. 1f, iii, iv and v) but not with *E. coli* harboring the vector control (Fig. 1f, ii). We conclude that the Mak proteins from *V. cholerae* are necessary and sufficient for the morphological changes observed in intestinal cells and other tissues of the worm.

To test whether bacterial accumulation in the intestines was the result of defecation defects, we examined expression of the defecation motor program (DMP). The DMP consists of a series of rhythmic muscular contractions normally occurring every 40–50 s^13^. The worms fed with *E. coli* Top10/pBAD18 showed a typical defecation rhythm with regular contractions of posterior body-wall muscles and regular expulsions with average defecation periods of 42.2 s (Supplementary Fig. 2A). Worms fed with *E. coli* expressing Mak proteins showed a severe defect in defecation. After 12 h, the contraction period for posterior body-wall muscles was extended with an average contraction period of 59.55 s (Supplementary Fig. 2B). These worms also showed an almost complete absence of the accompanying expulsion of the gut content.

Additional experiments analyzed endoplasmic reticulum (ER) and mitochondrial stress responses. A marker for ER stress, *hsp-4*::GFP, appeared weakly induced in worms exposed to *E. coli*/*makD*^*+*^*C*^*+*^*B*^*+*^*A*^*+*^ (Supplementary Fig. 2C, ii). Mitochondrial stress marker *hsp-6*::GFP appeared slightly induced (Supplementary Fig. 2C, iv) whereas another, *hsp-60*::GFP, was not induced (Supplementary Fig. 2C, vi).

As positive controls for stress induction we used three different RNAi clones to provoke stress in the worms: *pdi-2*(RNAi), *cyc-1*(RNAi), and *atp-5*(RNAi), which inhibit expression of protein disulfide isomerase, cytochrome c1, and ATP synthase subunit d, respectively. Such inhibition is known to induce expression of the ER and mitochondrial stress markers for the unfolded protein response (UPR)^14, 15^. There was a strong stress response in the worms treated with each of the the RNAi clones whereas in the worms subjected to Mak protein producing bacteria little or no induction of stress occurred (Supplementary Fig. 2D, ii, vi and ix). These observations suggest that bacteria expressing the *makD*^*+*^*C*^*+*^*B*^*+*^*A*^*+*^ operon do not kill *C. elegans* by targeting general functions of mitochondria or of the ER.

### Effect of *V. cholerae* MakA in zebrafish infection

Zebrafish can be used for studies of many different bacterial species^16–21^. We reasoned that studies of *V. cholerae* colonization within the intestinal tract of axenic zebrafish embryos and adult models would be relevant for characterization of potential virulence factors since these models support a natural route for gastrointestinal infections.

Zebrafish embryos showed a survival rate of 80% even after 12 h of incubation with 1 × 10^6^ bacteria per mL of *E. coli* Top10/vector control (Supplementary Fig. 3A). However, *E. coli* Top10 expressing either the *makC*^*+*^*B*^*+*^*A*^*+*^ or *makD*^*+*^*C*^*+*^*B*^*+*^*A*^*+*^ genes caused reduced survival with many dead embryos within 4–6 h of co-incubation and mortality of the majority within 9–12 h (Supplementary Fig. 3A). Enumeration of CFU of internalized bacteria showed high levels by the *E. coli* Top10/*makC*^*+*^*B*^*+*^*A*^*+*^ and *E. coli* Top10/*makD*^*+*^*C*^*+*^*B*^*+*^*A*^*+*^ bacteria in the zebrafish embryos (Supplementary Fig. 3B) and colonization was visualized by fluorescense microscopy after infection with derivatives co-expressing GFP (Supplementary Fig. 3C). We observed similar effects on adult zebrafish in presence of a 10 fold higher bacterial burden (1 × 10^7^ bacteria per mL) after 16–24 h incubation (Supplementary Fig. 3D). Expression of MakA in *V. cholerae* A1552 exhibited a similar effect on adult zebrafish and 50–100% were killed within 24–30 h (Supplementary Fig. 3D). Adult zebrafish survived up to 40 h in water containing 1 × 10^7^ bacteria per mL of vector control. Our results show that the *mak* operon of *V. cholerae* can cause toxicity to zebrafish.

### Flagella-mediated secretion of MakA protein

**Fig. 2:**
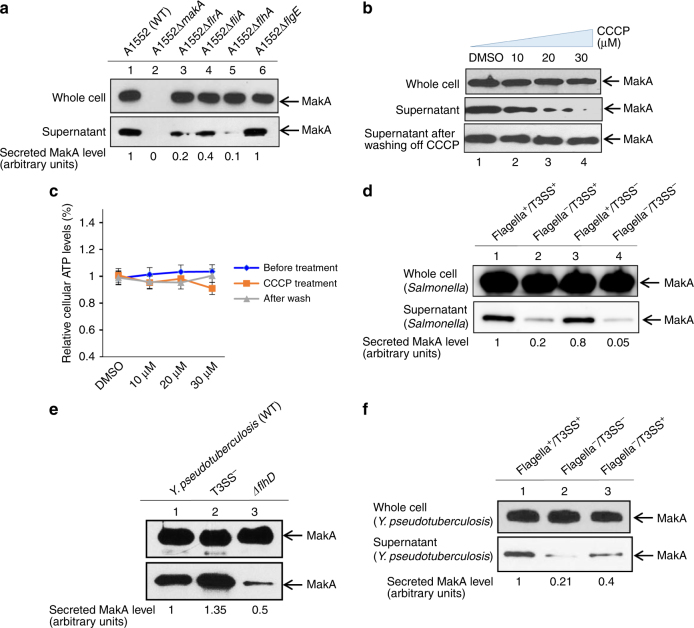
Role of bacterial flagellar machinery and proton motive force in secretion of MakA. **a** Immunoblot analysis of MakA secretion (lower panel) and expression (upper panel) of *V. cholerae* strain A1552 and its flagellar cascade mutants. Immuno-reacted band intensities relative to the level of secreted MakA from the wild-type strain set to 1.0 are shown; **b** MakA expression and secretion in A1552 grown in LB containing DMSO or 10, 20, 30 μM CCCP (upper and middle panels, respectively), restoration of secretion following washing with CCCP-free medium (lower panel); **c** ATP levels in A1552 treated with different dosage of CCCP; **d** Secretion and expression of MakA in *S*. Typhimurium and its mutant derivatives ectopically expressing *makA*: the wild type strain LT2 (Flagella^+^/T3SS^+^), lane 1; flagellar cascade mutant (Flagella^−^/T3SS^+^), lane 2; T3SS mutant ∆*spi*-1, ∆*spi*-2 strain (Flagella^+^/T3SS^−^), lane 3; both flagellar and T3SS cascade mutant (Flagella^−^/T3SS^−^), lane 4. Immuno-reacted band intensities relative to the level of secreted MakA from the wild-type strain set to 1.0 are shown; **e** Secretion and expression of MakA in *Y. pseudotuberculosis* and its mutant derivatives ectopically expressing *makA*: the wild type strain YPIII (lane 1), T3SS mutant (lane 2), Δ*flhD* (lane3). **f** Secretion and expression of MakA in *Y. pseudotuberculosis* grown under conditions favoring or inhibiting flagellar T3SS and/or T3SS expression. Band intensities of secreted MakA relative to the Flagella^+^/T3SS^+^ strain are shown

Using immunoblot analysis, we observed efficient secretion of MakA into the culture supernatant (Fig. 2a, lane 1 lower panel). Gram-negative bacteria may be equipped with different mechanisms to secrete effector proteins. For example, the PrtV protein is released by *V. cholerae* type II secretion system (T2SS)^22^ and the *V. cholerae* type VI secretion system (T6SS) is involved in protection of *V. cholerae* against predators^23^. Therefore, we tested if secretion of MakA was affected in T2SS and T6SS mutants of *V. cholerae*. The T2SS-deficient mutants, Δ*epsC* and Δ*epsG*, showed no detectable impairment in MakA secretion (Supplementary Fig. 3E, upper panel, lanes 5 and 6) although secretion of PrtV was abolished (Supplementary Fig. 3E, lower panel, lanes 5 and 6). Furthermore, MakA was efficiently secreted in the T6SS deficient Δ*vipA* and Δ*vgrG3* mutants (Supplementary Fig. 3F, upper panel, lanes 7 and 8), which are impaired for secretion of Hcp (Supplementary Fig. 3F, lower panel, lanes 7 and 8). *V. cholerae* O1 strain A1552 does not encode a virulence-associated T3SS.

Next, we tested the role of the flagellum in MakA secretion using mutants defective in flagellar assembly or function. Flagellar biosynthesis in *Vibrio* follows a strict hierarchial gene regulation^24^. On top resides FlrA, a regulator of early flagellar genes encoding basal body components and the flagellar T3SS, including the integral-membrane protein FlhA, responsible for the proton-motive force-driven secretion of flagellar building blocks^25^. The molecular ruler protein FliK^26^ measures the length of the hook and terminates hook FlgE protein polymerization at a final length of 55 nm (in *Salmonella*)^27^. Upon hook-basal-body completion, the flagellar T3SS switches substrate specificity to late substrates, including the anti-sigma factor FlgM, which prevents premature activation of the flagellar-specific sigma 28^28^. Surprisingly, secretion of MakA was reduced in three tested flagella regulator mutants, ∆*flrA*, ∆*fliA*, and ∆*flhA* whereas the whole cell level remained unaffected (Fig. 2a, lanes 3–5, upper and lower panels). Defective MakA secretion was most obvious in the ∆*flhA* mutant, a central component of the flagellar T3SS (Fig. 2a, lane 5, lower panel). In contrast, the ∆*flgE* mutant deficient in hook assembly did not seem to affect MakA secretion (Fig. 2a, lane 6, lower panel). It suggested that MakA might be recognized by the flagellar T3SS as an early secretion substrate.

A substantial level of MakA was detected in the supernatant of the Δ*fliA* mutant, consistent with the hypothesis that MakA is secreted as an early substrate (Fig. 2a, lane 4, lower panel). A presence of cytoplasmic proteins in the supernatant caused by cell lysis was excluded by immunoblot analysis against the cytoplasmic protein Crp (Supplementary Fig. 3G).

Reduced levels of MakA secreted by the *fliA* mutant may be due to positive feedback of σ28 to FlrA. A deletion of *fliA* might result in reduced FlrA expression, which in turn would reduce flagella synthesis and explain the reduced MakA secretion. Alternatively, it is possible that outer membrane vesicle-mediated secretion of MakA might occur in this case.

Translocation of flagella-associated proteins is mediated via the fT3SS situated at the base of flagella and driven by the proton motive force^29^. To ascertain the energy source involved, we analyzed the effect of the uncoupler protonophore CCCP. In comparison with *V. cholerae* A1552 treated with DMSO as control, MakA secretion in the presence of 10, 20, 30 μM CCCP for 30 min was proportionally reduced with almost no detectable secretion from the 30 μM CCCP-treated bacteria (Fig. 2b, middle panel, lanes 1–4). Total cellular ATP levels (Fig. 2c) indicated that 10–30 µM CCCP had only a small effect on overall cellular energy level as described before^29^. Likewise, the treatment did not affect total MakA level in the cells (Fig. 2b, upper panel) or the bacterial growth (Supplementary Fig. 3H). Furthermore, the effect of CCCP on secretion of MakA was reversible and normal secretion was largely restored following washout of the CCCP (Fig. 2b, lower panel).

### Secretion of MakA in Salmonella enterica serovar Typhimurium and Yersinia pseudotuberculosis

Many components of the flagellar systems are homologous in sequence or function in different bacterial species^30^. To test if MakA can be secreted by the fT3SS and/or vT3SS of other Gram-negative bacteria, the *makA*^+^ gene expressed from a P_BAD_-promoter, was introduced into the *S*. Typhimurium wild-type strain LT2 and its different mutant derivatives: the *∆fla* mutant (complete flagella null mutant), the ∆*spi-1*∆*spi-2* mutant (deletion of both injectisome loci) and the *spi-1* ∆*spi-2* ∆*fla* mutant (deleted for genes encoding for the flagellum and both injectisome devices).

The *S*. *enterica* serovar Typhimurium exported MakA efficiently (Fig. 2d, lane 1). Reduced MakA secretion was observed in the Flagella^−^/T3SS^+^ and the Flagella^−^/T3SS^−^ strains (Fig. 2d, lanes 2 and 4, lower panel) although the whole cell level was not affected (Fig. 2d, lanes 2 and 4, upper panel). The injectisome-deficient mutant ∆*spi-1* ∆*spi-2* strain (Flagella^+^/T3SS^−^) did not show reduced MakA secretion (Fig. 2d, lane 3). The *makA*^*+*^ construct was also introduced into wild-type YPIII, the T3SS and flagellum mutant *Y. pseudotuberculosis* strains. Secretion of MakA was clearly reduced in the flagellar mutant (Δ*flhD*) strain (Fig. 2e, lower panel, lane 3) whereas secretion was slightly increased in the T3SS mutant (Fig. 2e, lower panel, lane 2). Effector secretion via the vT3SS in *Y. pseudotuberculosis* can be modulated by Ca^2+^ and flagellar bio-synthesis can be controlled by bacteria grown in LB at 26 °C or 37 °C^31, 32^. We cultured *Y. pseudotuberculosis* strain YPIII constitutively expressing MakA under the above-mentioned conditions and analyzed secretion by immunoblotting. Secretion of MakA under flagella inducing conditions was more abundant than under conditions that prevented flagella formation and protein secretion via the vT3SS (Fig. 2f, compare lane 1 and 2, lower panel). These results suggested that flagella-mediated secretion of MakA may occur in *Y. pseudotuberculosis* YPIII as well. When *Y. pseudotuberculosis* was grown under a condition that suppressed flagella but allowed vT3SS expression, secretion of MakA was partially recovered indicating that MakA can be secreted via the vT3SS in the absence of the fT3SS at least in case of *Y. pseudotuberculosis* (Fig. 2f, lane 3).

### Polar localization of MakA at the flagellar base

**Fig. 3:**
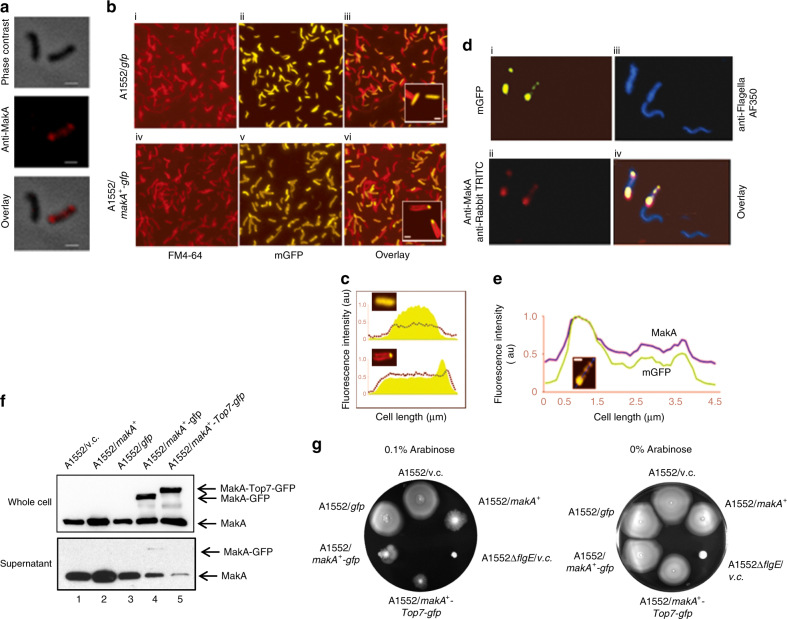
Polar localization of MakA in *V. cholerae*, immunoblot and motility assay of MakA and MakA chimeric proteins expressing *V. cholerae* strains. **a** MakA was visualized in A1552 cells using immunofluorescence with α-MakA antiserum: phase contrast image (i); focal localization of MakA (red) (ii); overlayed image (iii) scale bars, 500 nm; **b** Live-cell fluorescence microscopy of A1552/*mgfp* and A1552/*makA*^*+*^-*mgfp*: FM4-64 stained cells (i and iv); GFP distribution in A1552/*mgfp* (ii, iii and inset); polar localization of green fluorescence in A1552/*makA*^*+*^-*mgfp* (v, vi and inset); **c** fluorescence intensity pattern (vii) of A1552/*mgfp* (above) and (A1552/*makA*^+^-*mgfp* (below); scale bars, 3 µm; scale bars in the insets, 500 nm; **d** Immunofluorescence microscopy of A1552/*makA*^*+*^-*mgfp*: polar localization of GFP fluorescence (i); immunofluorescence showing polar localization of MakA (ii); immunofluorescence using AF350 labeled anti-flagella antibody (iii); overlay showing co-localization of MakA (red) with mGFP (green) at the flagellar pole (iv); **e** fluorescence intensity pattern of A1552/*makA*^+^-*mgfp* matches with that of MakA localization, suggesting that mGFP localization is an indicator of MakA position in cells (v). scale bars, 1 µm; **f** Immunoblot of MakA expression (upper panel) and secretion (lower panel) in *V. cholerae* A1552 harboring *makA* gene alone or chimeric *makA* constructs with *mgfp* and/or *Top7*; **g** Motility assay using the A1552/*makA*^*+*^, the A1552/*makA*^*+*^*-mgfp*, and the A1552/*makA*^*+*^*-Top7-mgfp* strains on LA plates with or without 0.1% arabinose induction

Prior to secretion, fT3SS substrates presumably need to be localized to the base of the flagellum. MakA localization was analyzed with immunofluorescence-phase contrast combination microscopy. As shown in Fig. 3a; panels ii and iii, we detected a more abundant localization of MakA near the bacterial poles. Next we constructed a C-terminal translational fusion of MakA to a modified green fluorescent protein (mGFP) and the resulting construct (A1552/*makA*^+^-*gfp*) constitutively expressed a MakA-mGFP chimeric protein. As shown in Fig. 3b, ii, iii and Fig. 3c (upper panel), fluorescence showed a uniform cell distribution when bacteria (A1552/*gfp*) expressed mGFP alone, whereas MakA-mGFP resulted in a striking fluorescence peak near one of the bacterial cell poles (Fig. 3b, v, vi and Fig. 3c (lower panel). These results demonstrated an inherent property of MakA to localize at the cell poles.

To visualize MakA and flagella simultaneously, bacteria expressing MakA-mGFP were subjected to immunofluorescence (IF) microscopy using antibodies labeled with AF-350 dye (α-Flagella-AF350). MakA-mGFP showed its characteristic focal polar localization (Fig. 3d, i). We confirmed presence of MakA in the mGFP foci by IF using α-MakA antiserum (Fig. 3d, ii). Immuno-stained flagella (blue) were observed either attached to, or detached from, the bacteria (Fig. 3d, iii). Fluorescence intensity of MakA was highest at the cell pole of the flagellum as indicated by the quantification shown in Fig. 3e. MakA therefore appeared most abundant at the flagellated cell pole (Fig. 3d, iv and e), near the base of the flagellum, supporting the idea that MakA may be exported via the flagellar channel.

### MakA passage through the flagellar channel

Secretion through the narrow central channel of the flagellum presumably requires active substrate unfolding as proposed by studies showing that GFP tagging of injectisome substrates interfered with their secretion^33^. We observed a similar phenotype with MakA-mGFP when expressed by *V. cholerae* (A1552/*makA*^+^-*mgfp*). Immunoblot analysis using α-MakA antibody showed that, despite its strong whole cell expression level (Fig. 3f, lane 4, upper panel), MakA-mGFP was almost undectable in the supernatant while secretion of native MakA was still detected although at a reduced level (Fig. 3f, lane 4, lower panel). In contrast, the A1552/*makA*^*+*^ showed efficient secretion of MakA (Fig. 3f, lane 2, lower panel). These observations agreed with our hypothesis that MakA is an fT3S substrate and that GFP tagging might prevent secretion. Our hypothesis was further corroborated by motility retardation in strain A1552 expressing MakA-GFP (Fig. 3g and Supplementary Fig. 4A). Interestingly, a higher expression level of MakA also imparted partial motility impediment. There was no apparent alteration in motility among the strains when the expression of MakA or MakA-mGFP was not induced with arabinose (Fig. 3g). We thus concluded that the impaired motility phenotype in A1552/*makA*^+^ could be due to disproportionate occupancy of the fT3S machinery by MakA, leading to competitive inhibition of secretion of fT3SS substrates needed for flagellum assembly.

We next tried to visualize the presumed transit of MakA through the flagellar channel. In a recent study, Dohlich et al. successfully visualized substrate transition through the T3S channel in *Shigella*, by tagging the protein IpaB with a bulky methyltransferase, RrmA, which has a highly stable trefoil ‘Knot’^34^. We employed a similar strategy and used a small artificial globular protein, Top7, which folds into an unconventional and highly stable helical coiled coil structure not found in nature^35^, to engineer a hybrid gene construct, *makA-Top7-mgfp*. After induction of high level expression of MakA-Top7-mGFP in *V. cholerae* strain A1552, we did not detect secretion of the chimeric protein and we observed much reduced levels of secretion of native MakA, although the whole cell expression level was not changed (Fig. 3f, lane 5 upper and lower panels), implying an entrapment of the chimeric protein in the narrow central channel of flagellum. Besides, over-expression of MakA-Top7-mGFP also greatly impaired the bacterial motility (Fig. 3g and Supplementary Fig. 4A). The impaired motility phenotype was also observed in *S*. Typhimurium strain LT2 (Supplementary Fig. 4B and C).

We used a combination of fluorescence and IF microscopy to directly observe trapping of the MakA-Top7-mGFP protein inside the flagellar channel. The C-terminal mGFP tag appeared as a prominent blob at the bacterial pole. Likewise, α-MakA-mediated IF also revealed a spotted pattern of strong MakA signals co-localized with mGFP (Supplementary Fig. 5A). By immuno-fluorescence analysis we could not detect any distally translocated MakA domains within the lumen of the flagellum, presumably due to shielding of MakA epitopes by rigid flagellar sub-structures. The calculated contour length of unfolded 396 amino acid polypeptide MakA (140 nm) would not be able to span the entire length of wild-type flagella, which grow up to several micrometers in length^36^. Therefore, we expressed MakA-Top7-mGFP in a *V. cholerae* hook mutant, A1552Δ*flgE*. The Δ*flgE* mutant is non motile (Fig. 3g and Supplementary Fig. 4A). Nonetheless, it maintains a functional flagellar basal body which actively exports fT3SS specific substrate proteins^37^. We observed a prompt secretion of native MakA in the A1552Δ*flgE* strain (Fig. 2a, lane 6, lower panel and Supplementary Fig. 5B, lane 1, lower panel). Co-expression of MakA-Top7-mGFP caused a substiantially reduced secretion of native MakA in the A1552Δ*flgE* mutant (Supplementary Fig. 5B, lane 4, lower panel). Analysis of mGFP fluorescence revealed a polar localization of the fusion protein (Supplementary Fig. 5C, i). We detected co-localization of MakA and mGFP at the cell poles, and a noticeable proportion of MakA epitopes was observed as fluorescent spikes outside both the mGFP foci and cell periphery (Supplementary Fig. 5C, i, ii and iv). We concluded that these prominent MakA spikes, which were separated from their cognate mGFP (and Top7) by the cell envelope, represent distal ends of the MakA-Top7-mGFP chimera with its proximal unfolded end anchored at the base of the fT3S channel.

**Fig. 4:**
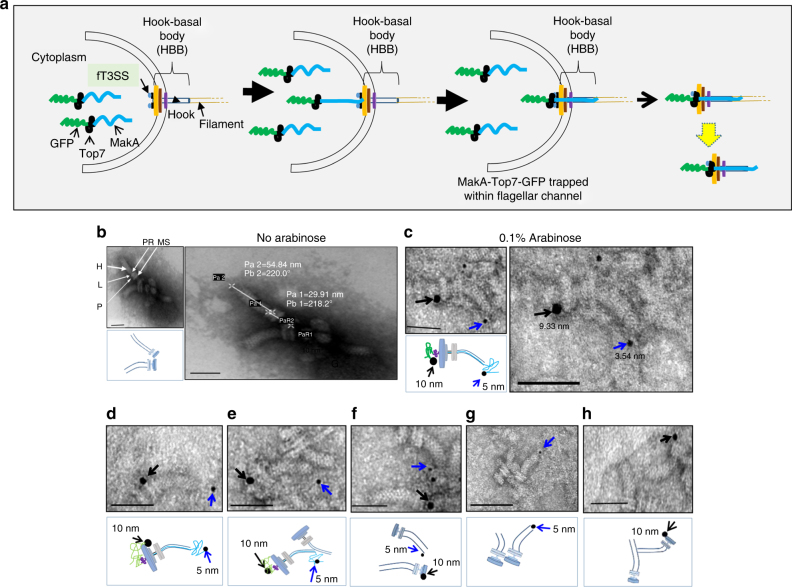
Transmission electron micrographs (TEM) of flagellar hook-basal body (HBB) complex (**a**) Schematic drawing showing localization of MakA-Top7-mGFP chimeric protein within the flagellum of the bacteria (**b**) TEM analysis of HBB complexes isolated from *V. cholerae* A1552/*makA*^*+*^*-Top7-mgfp* without arabinose induction. H, hook; L, L-ring; P, P-ring; PR, proximal rod; MS, MS-ring; (**c**-**h**) TEM analysis of immunogold labeled HBB complex purified from A1552/*makA*^*+*^*-Top7-mgfp*. Immunolocalization of proximal MakA and distal GFP proteins was performed using anti-MakA and anti-GFP antibodies and detected with α-Rabbit-5 nm (red arrows) and anti-Mouse-10 nm (green arrows) gold conjugated antibodies, respectively. Sketch below each micrograph explains the possible orientation of exposed epitopes of the MakA-Top7-mGFP chimera with respect to the HBB. Scale bars, 55 nm

Next we isolated flagella hook-basal body (HBB) complexes without and with entrapped MaKA-TOP7-mGFP inside the the flagellum channel (Fig. 4b, c). Localization of the protein is illustrated schematically in Fig. 4a. To monitor the association of MakA proteins with the HBB complexes, we performed immunoelectron microscopic analyses using anti-MakA antiserum and anti-GFP antibody coated on 5 and 10 nm gold particles, respectively. We detected the 5 nm gold particles (blue arrows) at the end of the hook and 10 nm gold particles (black arrows) at the base of the HBB complexes (Fig. 4c to h). The results strongly support our conclusion that the MakA protein indeed was secreted by passage through the central secretion channel of the *V. cholerae* flagellum.

### A conserved gate-recognition motif in MakA secretion

To enter the export pathway, MakA protein synthesized in the cytoplasm must be targeted to the membrane export machinery of the fT3SS. A conserved surface-exposed hydrophobic gate-recognition motif (with sequence Fxxxϕ, where ϕ is any hydrophobic residue) at the N-terminal 30–50 residues of rod and hook proteins was shown to be required for recognition by the flagellar T3SS^38^. Interestingly, we observed a potential hydrophobic (FTPP) motif at N-terminal 37–40 amino acid residues of the MakA protein. To test if the FTPP motif affects MakA secretion, we substituted the hydrophobic phenylalanine residue with hydrophilic aspartic acid. The DTPP mutant clone *makA*^(F37D)^ was introduced into the A1552Δ*makA* strain and secretion was examined. As shown in Supplementary Fig. 6A, lane 4, the secretion of MakA^(F37D)^ was dramatically reduced in comparison with the wild-type MakA (Supplementary Fig. 6A, lane 3). To examine the subcellular localization of MakA^(F37D)^, we constructed a plasmid expressing MakA^(F37D)^-mGFP fusion protein. The GFP-fluorescence showed a uniform distribution in most cells when bacteria expressed MakA^(F37D)^-mGFP, whereas wild-type MakA-mGFP showed a predominant polar localization (Supplementary Fig. 6B, compare panels viii and ix with panels v and vi). These results support the hypothesis that the FTPP motif is essential for MakA export via the fT3SS.

### Structural analysis of MakA protein

**Fig. 5:**
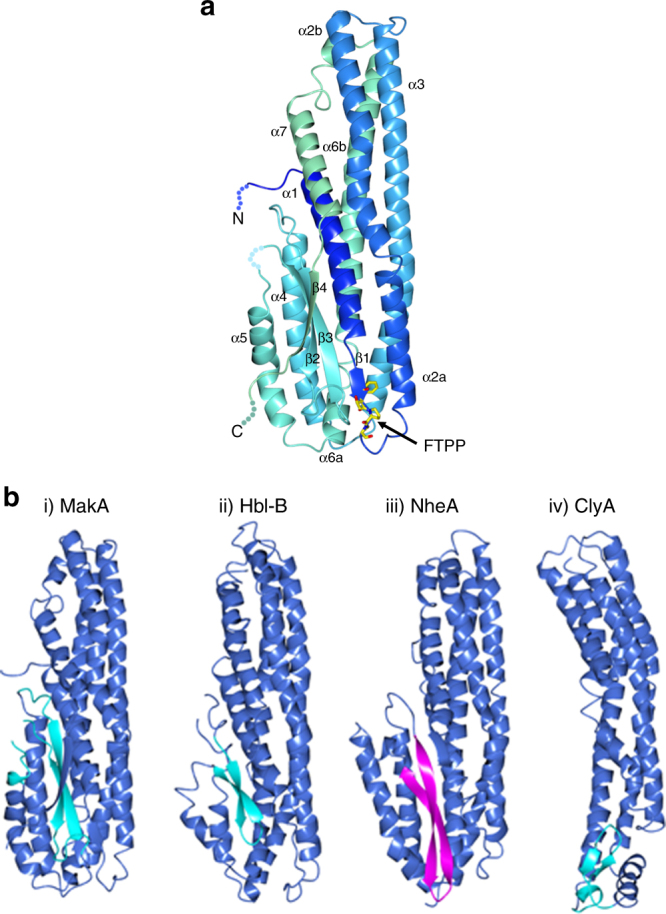
Overall structure of MakA and comparison of MakA with related structures. **a** A ribbon presentation of MakA where the coloring blends through the model from blue (N-terminus) to red (C-terminus). The tail subdomain comprises helices 1, 2, 3, 6 and the head subdomain comprises helices 2 and 5 and a β-sheet. The flexible regions that have not been modeled are depicted with dotted lines. The side putative gate-recognition motif (FTTP) is illustrated as stick models and indicated with an arrow. The head domain comprises helices 4 and 5 and a β-sheet. **b** Ribbon diagram of (i) MakA, (ii) Hbl-B^40^ from *B. cereus*, (iii) NheA^41^ from *B. cereus* and (iv) the soluble form of ClyA^42^ from *E. coli*. The structures of MakA, Hbl-B, NheA, and ClyA are depicted in blue and the hydrophobic β-strands of MakA, Hhl-B, and ClyA are illustrated in green. The amphiphatic, but structurally equivalent β-strands of NheA are depicted in pink

To analyze the structure of MakA, we generated crystals of the purified protein and performed X-ray diffraction studies. MakA can be described as a two-domain protein, a tail and a head (Fig. 5a). The tail consists of a bundle of five long helices (helix 1, 2, 3, 6, and 7). α1 ends in a short β-strand, β1. α2 is split in two parts, α2a and α2b, after a kink at W64. The second part, α2b, partially adopts 3_10_ character. α6 is also split in two parts, α6a and α6b, separated by a short 3_10_ helix (residues 274–278). The head domain consists of helix 4 and 5 and three β-strands that together with the β1-strand of the tail domain forms a four-stranded sheet (strand order β1β3β2β4). Overall, the structure is well-defined except for three regions with disordered electron density: the first five residues from the N-terminus, the last four from the C-terminus and the loop connecting β3 with α5, (residues 235–239). A Dali search^39^ with MakA finds two bacterial toxins with high structural similarity (rmsd ~3.2 and a Z-score higher than 21.5): hemolysin BL, HBL-B, and the non-hemolytic enterotoxin, NheA from *Bacillus cereus* (PDB codes 2NRJ and 4K1P)^40, 41^. The soluble form of ClyA from *E. coli* (Z-score 9.8, r.m.s.d. 5.2 on 226 aligned residues^39^) is the best characterized bacterial toxin of this superfamily (PDB code 4PHQ)^42^. Comparison of MakA with HBL-B, NheA and ClyA is shown in Fig. 5b. Their sequence identity is 11, 14, and 6 %, respectively. Like MakA, HBL-B and NheA have their head subdomains turned upwards, packing against the tail helices^40, 41^. ClyA, on the contrary, has its equivalent to the head subdomain turned downwards resulting in an elongated tail subdomain^43^. The FTPP motif in MakA is located on the short β1-strand and the loop that connects it to α2. Interestingly, F37 is not directly exposed but stacks with F47 in the beginning of α2. F47 is in turn stacking with the third residue of the motif, P39. Thus the motif is likely to be important for rigidifying the loop between β1 and α2.

## Discussion

In this study, we identified a novel virulence factor of *V. cholerae*, the secreted MakA protein encoded by the *mak* operon, and demonstrated its role in lethal bacterial infections of *C. elegans* and zebrafish.

Characterization of MakA transport from the bacteria revealed that it occurred by flagellum-dependent secretion. Energy for translocation through the membrane seems to be provided, at least in part, by the proton motive force as we showed that MakA secretion was inhibited by CCCP. Furthermore, we visualized secreted MakA trapped within the flagellar export channel and showed that a conserved N-terminal FTPP motif is essential for MakA secretion, presumably acting as a hydrophobic gate-recognition motif, similar to the N-terminal motif described for flagellar substrates of *Salmonella*^44^. For subunits of the flagellar filament, hook–filament junction, and filament cap, specific chaperones appear to be critical for targeting to the membrane export machinery^38, 45^. It remains to be investigated if any chaperone is required for MakA export. Our investigation of MakA secretion revealed a mechanism that represents a hitherto undiscovered secretion system of virulence proteins in *V. cholerae*. The *V. cholerae* flagellum has so far not been implicated as a secretory machine for any of its toxins or other proteins not required for motility. Presumably, many of the virulence factors that bacterial pathogens may display during infection of human hosts have evolved in the natural ecological niches^3^.

Analysis of the MakA sequence to predict transmembrane domains (TMHMM server)^46^ predicts two transmembrane helixes (residues 198–220 and 224–246). This region mainly consists of two β-strands, β2 and β3, that are integrated in the head domain β-sheet, with the sequence VGIFMLVAGVALTVATAGTAAAVSGGIAAVGAA. The soluble, monomeric form of ClyA has a hydrophobic β-tongue with sequence AAAGVVAGPFGLIISYSIAAGVV, in a similar position^43^. Similarly, HBL-B has hydrophobic β-strands with the sequence GAILGLPIIGGIIVGVA, in equivalent position as MakA. HBL toxin is composed of three distinct protein components, B, L1, and L2 which together possess cytotoxic activities^47^. However, no evidence indicates that HBL-B alone can rearrange and form a pore without proteins L1 and L2^41^. In NheA, the corresponding β-strand has a more amphiphatic character (IIKGSINIGKQVFTITNQTAQTKTIDFVSIGT) but like the HBL toxin, Nhe is a tripartitie enterotoxin requiring three proteins that together function as a pore-forming toxin^48^. Based on gel filtration analysis, MakA is undoubtedly monomeric in solution and pore-forming activity has not been detected.

Defecation in *C. elegans* is a rhythmic behavior that is regulated by a Ca^2+^ oscillation generated in the cells of the intestine by activation of the inositol 1,4,5-trisphosphate receptor^49, 50^. We suggest that fluctuations of Ca^2+^ levels in *C. elegans* intestine caused by MakA might account for the observed impairment of the DMP.

The discoveries with MakA highlight a non-conventional and direct role of flagella in pathogenesis of *V. cholerae*. While MakA presumably evolved with a primary role in the naturally occuring predator interactions of *V. cholerae* in its aquatic environments, our findings suggest that it should be considered also as a factor potentially contributing to pathogenesis in mammalian infections.

## Methods

### Bacterial strains and growth conditions

All bacterial strains are listed in Supplementary Table 1. *Vibrio cholerae*, *Salmonella enterica* serovar Typhimurium, and *Escherichia coli* strains were grown in LB broth or on 1.5% LB agar at 37 °C. *Yersinia pseudotuberculosis* strains were grown at 26 °C in LB broth or on 1.5% LB agar and at 37 °C in LB broth supplemented with 2.5 mM CaCl_2_. For tests with *C. elegans* (see below), bacteria were cultured on nematode growth medium (NGM) agar plates^51^. The following antibiotics were used: 100 µg/mL carbenicillin, 50 µg/mL kanamycin or 100 µg/mL rifampicin. For strains harboring expression plasmids, gene expression was induced by adding 0.1% or 0.05% L-arabinose.

### *Caenorhabditis elegans* infection survival assay and intestinal morphology

*C. elegans* strains N2 (wild type), SU93 (*jcIs1* [*ajm-1::GFP*]) and WS4918 (*opIs310* [*act-5*::*YFP*]), were provided by the Caenorhabditis Genetics Center (CGC). *C. elegans* killing assays were performed as described earlier^3^. Briefly, an overnight LB broth culture (100 μL) of the test bacterial strain was spread on a 5-cm-diameter nematode growth medium agar plate and incubated at 37 °C for 16 h before the bacterial lawn was seeded with 50 L4 stage worms of *C. elegans* WT strain Bristol N2. Plates were incubated at room temperature (23 °C) and scored for live worms every day. A worm was considered dead when it no longer responded to touch. Multiple experiments (three to four worm-killing assays per bacterial strain) were done, and the data presented are from a representative experiment. Intestinal morphology of live worms was examined by Nomarski differential interference contrast (DIC) microscopy (Leica DMIRB microscope) before and after infection. Fluorescence confocal microscopy of immobilized worms was performed using a Nikon A1 microscope and analyzed using NIS-Elements AR 4.30.02 software.

### Defecation cycles of *C. elegans*

For recording of defecation rhythm of *C. elegans*, the worms fed with *E. coli* Top10/vector control or *E. coli* Top10/*mak*^*+*^*D*^*+*^*C*^*+*^*B*^*+*^*A*^*+*^ as mentioned above for 12 h, were individually transferred to fresh plates containing corresponding bacterial strains and allowed to settle for 15 min. Muslce contractions in the defecation cycle in individual worms were observed over a period of 10 min. The posterior body-wall muscle contractions and expulsion muscle contractions were recorded as described previously^52^. Briefly, for recording of defecation rhythm of *C. elegans*, the worms fed with *E. coli* Top10/vector or *E. coli* Top10/*makD*^*+*^*C*^*+*^*B*^*+*^*A*^*+*^ for 12 h and then worms were individually transferred to fresh plates containing corresponding bacterial strains and allowed to settle for 15 min. Movement of individual worms were observed over a period of 10 min. The posterior body-wall muscle contractions and expulsion muscle contractions were recorded.

### *C. elegans* ER and mitochondrial stress response assays

25 fourth larval stage (L4) hermaphrodite worms carrying a stress marker were placed onto nematode growth medium (NGM) plates containing either the control *E. coli* strain (*E. coli* Top10/vector control) or the strain expressing all four Mak proteins (*E. coli* Top10/*makD*^*+*^*C*^*+*^*B*^*+*^*A*^*+*^). The induction of stress marker expression was assessed by fluorescence microscopy after 48 h. The genotypes of the worms used were *zcIs4*[*hsp-4*::GFP]^53^, *zcIs13[hsp-6*::GFP]^54^ and *zcIs9*[*hsp-60*::GFP]^54^. For comparison, in separate cultures, stress was induced in worms harboring the transgenes by RNAi of the *pdi-2*, *cyc-1*, and *atp-5* genes, respectively.

### Zebrafish (*Danio rerio*) infection assay

All zebrafish work was conducted in accordance with relevant European Union regulations for animal experimentation (Umeå regionala djurförsöksetiska nämnd Dnr A13–15).Maintenance and breeding of zebrafish: Zebrafish were maintained by standard procedures in the Umeå University Zebrafish Facility. We used15 adult male WT fish (3–4 months old) or 25 5–8 day old embryos/juveniles from WT fish (*Danio rerio*, London wild type [LWT]) were used. Adult male fish were paired in breeding chambers over night to induce spawning. Eggs were collected and placed in E3 medium (5,0 mM NaCl, 0,17 mM KCl, 0,33 mM CaCl_2_,33 mM MgSO4 with optional 1 p.p.m. methylene blue) at 28 °C until used in subsequent experiments. Embryos were staged as described previously^55^. For experiments with bacteria the E3 medium, without methylene blue, was supplemented with 1/20 LB medium in order to sustain bacterial gene expression.

Zebrafish embryo survival assay: 5-day old zebrafish embryos were anesthetized with 1% tricaine methanesulfonate (Sigma-Aldrich) and washed three times in fresh E3 buffer without methylene blue. Using Pasteur pipettes 10 healthy embryos were placed into each of several 90 mm petri dishes containing 20 mL fresh E3 medium and starved overnight at 27 °C. The *E. coli* strains harboring either vector control or desired expression plasmids were grown until OD 1.0 with or without addition of arabinose to induce the expression of cloned genes. Bacterial cells were washed once in E3 medium containing 1/20 × LB. Bacterial strains were added at a final concentration of 10^6^ CFU/mL into each plate containing 10 starved embryos and appropriate antibiotics with or without arabinose. Control plates contained the same components except bacteria. Plates were incubated at 27 °C for 2–24 h. Survival of zebrafish embryos was visually monitored at 2 h intervals. Embryos essentially unresponsive to touch were considered dead and at each time point, the number of live embryos was recorded. To determine the extent of bacterial colonization, live embryos in each plate were euthanized. Both dead and euthanized embryos were washed 3 times in 1 × PBS and treated with 50 µg/mL of gentamycin for 1 h at room temperature to kill adhered bacteria. Gentamycin treated embryos were washed three times with 1× PBS and homogenized using Dipomix homogenizer (VWR) at 2500 r.p.m. for 60 s. Subsequently, the CFU count was determined by serial dilution.

For microscopic visualization of colonization in fish embryo, 5-day old fish embryos were infected with *E. coli* Top10 derivatives strains harboring a green fluorescent protein expression plasmid alongside desired recombinant constructs cloned in vectors with replicon compatibility. Infection was done essentially as mentioned above using 10^6^ CFU/mL bacterial cells. After 9 h infection, live embryos were washed three times with 1× PBS and anesthetized in tricaine methanesulfonate. Individual embryos were mounted on 1% agarose stabs in 1× PBS, pH 7.4 and immediately analyzed using a Nikon Eclipse 90i upright microscope. Images were captured by a black and white 12 bit Hamamatsu OCRA-ER Digital camera using software NIS-Elements AR. Image data-analysis was done using NIS-Element AR 3.2.

For adult zebrafish infection, 1 × 10^7^ CFU/mL bacterial suspension was inoculated into 200 mL of tank water (distilled water with sea salt to reach a conductivity of 700 µs/cm) in a 500 mL beaker with perforated lid. LB was added to each beaker at a final dilution of 1/20 with appropriate antibiotics and arabinose concentrations. Four adult zebrafish (3 months old) were then transferred into each beaker and placed at 27 °C. Survival of fish was monitored at 2 h interval.

### Bacterial motility assay

Bacterial motility was tested on a semi-solid LA plate containing 0.25% agar by inoculating ~ 3 µL (10^7^ cells) overnight bacterial culture^29^. After 4–8 h incubation at 37 °C, the diameter of the motility swarms was measured and motility was calculated relative to the wildtype control.

### DNA manipulation

Oligonucleotide primers used are listed in Supplementary Table 2. PCR amplification was performed using Phusion DNA polymerase (Thermo Scientific) according to manufacturer’s recommendation. In-frame gene deletion in *V. cholerae* was performed as described^56^. Briefly, a 500-bp 5′ flanking sequence of the gene, including several nucleotides of the coding region, was PCR amplified with the primers described in Supplementary Table 2. The two PCR products were annealed at their overlapping region and amplified by PCR to produce a single DNA fragment, using the outer primers. The resulting PCR product, lacking most of the coding sequence of the gene, was digested with *XbaI* enzyme and ligated into a similarly digested pCVD442 suicide plasmid. pCVD442 containing mutant construct was introduced into *E. coli* SM10λpir by electroporation. The donor, *E. coli* S10λpir containing the plasmid pCVD442 containing mutant construct, was used for conjugal transfer to rifampin-resistant *V. cholerae* O1 El Tor A1552. A mixture containing equal volumes of the donor and recipient in LB was incubated for 6 h at 30 °C. The exconjugants were selected by plating the suspension onto LB plates supplemented with 100 μg/mL of rifampin and 100 μg/mL of carbenicillin (Cb) at 30 °C. After selection of the desired transconjugants on rifampin-plus-Cb plates, they were streaked on LB plates with 10% sucrose at 30 °C. Several colonies were purified from the plates, tested for Cb sensitivity, and then analyzed for the deletion using colony PCR.

For complementation of various null mutations with a corresponding wild type allele, as well as for ectopic expression of selected genes in different test strains, coding DNA sequences of individual target gene or set of genes were PCR amplified from A1552 genomic DNA and cloned into the plasmid pBAD18 at *SacI/XbaI* sites. Positive clones were confirmed by sequencing and introduced into *V. cholerae*, *E. coli*, *S. enterica* serovar Typhimurium or *Y. pseudotuberculosis* using electroporation. Plasmid pBSB269 was constructed by introducing a P_BAD_ promoter and a *gfp* gene by in vitro homologous recombination (In-Fusion® HD Cloning Kit, Clontech). Plasmid pBSB268 was generated by modifying the *gfp* gene to cause the amino acid substitution A206K^57^ in the plasmid pBSB269 and it thereby expresses monomeric GFP (mGFP) under control of the P_BAD_ promoter. Plasmid pBSB454 was generated to create a hybrid gene resulting in a C-terminal translational fusion protein of MakA and mGFP expressed from the P_BAD_ promoter. Plasmid pMDB113 was constructed to express a chimeric recombinant with an irreversibly folded Top7 synthetic protein sandwiched between N-terminal MakA and C- terminal mGFP. The *Top7* gene was introduced between *makA* and *mgfp* of pBSB454. Construction of *makA*(F37D) mutant clones was performed using either *pmakA*^*+*^ (pMDC004) or *pmakA*^*+*^*-mgfp* (pBSB454) as templates.

### Cloning, overexpression, and purification of MakA

The *makA* gene (UniProt accession code Q9KL64) was PCR amplified from genomic DNA of *V. cholerae* strain A1552 using forward primer KPC001 and reverse primer KPC002. The PCR product encoding residues 1–369 was digested with *NcoI* /*Acc65I* and cloned into pET-His1a, in-frame with a cleavable histidine affinity tag with the sequence MKHHHHHHPMSDYDIPTTENLYFQGAM. Since the introduction of an NcoI site in the 5´primer causes a mutation it was designed so that the initial MakA residue, Met, was mutated to Gly keeping the sequence from the second residue intact. After cleavage with TEV protease the final protein had the sequence GAMG followed by MakA residues 2–369. The construct was expressed in *E. coli* BL21(DE3) in LB broth supplemented with 50 μg/mL kanamycin. Induction was initiated with 0.5 mM isopropyl 1-thio-β-d-galactopyranoside (IPTG) at OD600nm 0.6 followed by growth for 5 h at 25 °C. Cells were harvested by centrifugation and the pellet stored at −80 °C until further use. The cell pellet was resuspended in 50 mM Tris-HCl pH 7.6, 0.3 M NaCl and 10 mM imidazole (lysis buffer) supplemented with 1% triton-X100 and sonicated on ice. The lysate was centrifuged at 39,000×*g* for 40 min. The supernatant was loaded onto a column packed with Ni-NTA agarose (Qiagen). The column was washed with 50 mM Tris-HCl pH 7.6, 0.3 M NaCl and 30 mM imidazole after which the protein was eluted with buffer containing 0.3 M imidazole. The buffer of the eluate was exchanged by dialysis to lysis buffer and incubated with 1% (w/w) TEV protease overnight. The cleaved protein was purified on the Ni-NTA agarose column again and the flow through and wash fractions were concentrated by an Amicon Ultra centrifugal filter device (Millipore). The protein was further purified on a HiLoadTM 16/60 SuperdexTM 200 prep-grade column (GE Healthcare) equilibrated with 20 mM Tris-HCl pH 7.6, 0.2 M NaCl. Fractions containing the peak of interest were concentrated in 20 mM Tris-HCl pH 7.6. Selenomethionine (SeMet)-labeled MakA was produced by growing the culture in M9 media supplemented with glucose at 37 °C. At an OD600 ~0.4, 100 mg/L each of lysine, threonine, phenylalanine and 50 mg/L each of leucine, isoleucine, valine, proline, SeMet were added and protein expression was induced with IPTG^58^. The SeMet-labeled protein was purified as described above.

### Crystallization and structure determination

Initial crystallization trials were performed at 20 °C using 6.5 mg/mL protein by sitting-drop vapor-diffusion in 96-well MRC-crystallization plates (Molecular Dimensions). Droplets of 0.5 µL protein solutions were mixed with equal volumes of screening solutions from Molecular Dimensions. The final crystallization condition was optimized to 0.2 M sodium acetate, 24% (w/v) PEG 8000, 0.1 M sodium cacodylate pH 6.5. Crystals of SeMet-labeled MakA were obtained in similar conditions.The crystals were soaked for 30 s in mother liquor solution supplemented with 15% (v/v) glycerol before they were flash cooled in liquid nitrogen and stored until data collection. Diffraction data were collected on a Pilatus3 2 M detector at beamline ID30A-1 at the European Synchrotron Radiation Facility, Grenoble, France. Diffraction images were processed with XDS^59^ and scaled with Aimless^60^ from the CCP4 program suite^61^. The structure of SeMet-labeled MakA was solved with SAD-phasing using AutoRickshaw^62^.

Density modification and automatic model building were performed using AutoRickshaw and ArpWarp^63^ and refined using phenix.refine^64^. The model was further built using rounds of manual building in COOT^65^. The SeMet-structure was used as a starting model for the refinement of native MakA and was refined as described above. Figures were prepared with CCP4mg^66^. Relevant processing and refinement statistics are summarized in Supplementary Table 3.

### SDS-PAGE and Western blot analysis

SDS-PAGE and immunoblotting was performend according to standard procedures^67^. Briefly, samples of bacterial cultures were harvested by centrifugation at 10,000×*g* to separate cells from the culture supernatant. Cell pellets were suspended in appropriate volume of 1 × Laemmli buffer and boiled to obtain whole cell lysates. Residual culture supernatants were filtered through 0.45 µm PVDF syringe filters (Millipore, USA) and subjected to protein precipitation with 10% trichloroacetic acid (TCA). Precipitated proteins were collected by centrifugation at 15,000×*g* for 15 min at 4 °C and dissolved by boiling for 5 min in 1 × Laemmli buffer. Protein samples were resolved on a SDS-PAGE and processed for immunoblotting. HRP-conjugated donkey anti-rabbit IgG (Agrisera AB, Sweden) was used as a secondary antibody. Detection was performed using Clarity® western ECL substrate (Bio Rad). Pre-stained protein molecular weight standards (SM0679, Fermentas) were used to determine the protein sizes. HRP-conjugated donkey anti-rabbit IgG 1:10,000 dilution (Agrisera AB) was used as a secondary antibody. Detection was performed using Clarity® western ECL substrate (Bio Rad). Protein molecular weight standards (SM0679, Fermentas) were used to determine the protein sizes.

### MakA secretion inhibition assay using CCCP

To test the effect of Cyanide m-chlorophenylhydrazone protonophore (CCCP) on the MakA protein secretion by *V. cholerae*, bacteria were grown in 5 mL LB at 37 °C until OD_600nm_ = 1.0. 1 mL culture was harvested in a microcentrifuge to collect the cell pellet. After washing the pellet with LB containing either DMSO or 10, 20, 30 μM of CCCP in DMSO, cell pellets were resuspended in the same medium and incubated at 37 °C for 30 min. Cultures were centrifuged at 12,000×*g* for 5 min. Both supernatants and pellets were then processed for immunoblotting to detect MakA. For the recovery experiment, following CCCP exposure cell pellets were collected by centrifugation and washed with LB and incubated again at 37 °C for 1 h.

### ATP Level analysis

Intracellular ATP levels in the *V. cholerae* A1552 treated with CCCP or DMSO control were measured using the bioluminescence-based time stable ATP determination kit (Proteinkinase.de) according to the manufacturer’s recommendations. Briefly, cells were harvested from 100 μL of bacterial cultures and immediately frozen at −20 °C. Pellets were then rapidly thawed, resuspended in 100 μL of lysis buffer and heated at 95 °C for 5 min. Equal volumes of cell lysate and ATP assay reagents were mixed in a microtiter plate and incubated at room temperature for 5 min. Luminescence was recorded using an Infinite M200 microplate reader (Tecan).

### Live cell and immunofluorescence microscopy

For live cell microscopy, bacteria were grown in LB media at 37 °C until OD_600nm_ 0.5, and the expression of genes was induced using 0.1% arabinose for 1 h. 100 μL of cell suspension was stained with 0.1 μg/mL of membrane dye, FM4-64 (FM®4-64FX(N-(3-triethylammoniumpropyl)-4-(6-(4-(diethylammino)phenyl)-hexatrienyl)-pyridiumdibromide; Invitrogen) for 15 min at room temperature. 10 μL of FM4-64 stained bacterial cells were placed on a 1% agarose slab and covered with a cover glass. For immunofluorescence microscopy bacterial cells were spread on a glass coverslip coated with 0.1% poly-L-lysine, air-dried and fixed in 2% paraformaldehyde. Fixed cells were permeabilized with 2 mg/mL lysozyme in GTE (0.9% glucose, 20 mM Tris HCl pH 8 and 10 mM EDTA) buffer for 30 min at 37 °C, washed with PBS and kept in 2% bovine serum albumin (BSA) in PBS for 1 h. To examine MakA localization, cells were incubated with α-MakA antiserum (Agrisera AB) diluted 1:5000 in BSA overnight at 4 °C, washed with PBS, incubated with either α-Rabbit-TRITC goat IgG antibodies (Jackson Immuno Research Laboratories; diluted 1:1000 in 1% BSA) or α-Rabbit-AF350 donkey IgG antibodies (Life Technologies; diluted 1:1000 in 1% BSA) for 1 h at room temperature, and washed with PBS. For simultaneous localization of flagella, cells were incubated with α-flagella-AF350 antiserum for 1 h at room temperature, followed by washing with PBS. Coverslips with stained cells were mounted on a glass slide treated with Dako Fluorescent Mounting Media with 0.1 μg/mL of membrane stain FM4-64. Images for both live cell and immunofluorescence microscopy were acquired using a Nikon Eclipse 90i motorized upright microscope. Image analysis was performed using the NIS-Element AR 3.2 software.

### Purification of flagellar hook-basal body (HBB) complex from bacteria

HBB complexes from *V. cholerae* were isolated using the method described previously by Aizawa and collegues^68^. Briefly, bacterial cells were harvested from 500 mL of late logarithmic phase cultures, and resuspended in 30 mL ice-cold sucrose solution (0.5 M sucrose, 0.1 M Tris-HCl [pH 8.0]) containing 1 mg/mL lysozyme and 10 mM EDTA (pH 8.0). The mixture was incubated at 4 °C for 30 min and lysed with 3 mL of 10% Triton X-100 followed by 3 mL of 0.1 M MgSO4. After incubation at 4 °C with gentle stirring for 2 h to obtain complete lysis, 3 mL of 0.1 M EDTA (pH 11) was added. The lysate was clarified by low speed centrifugation at 5000×*g* for 10 min and was adjusted to pH 11 with 5 N NaOH. The lysate was then centrifuged at 100,000×*g* for 60 min and a resulting pellet was resuspended in alkaline sucrose solution (10% sucrose, 0.1 M KCL, 0.1% Triton X-100 [pH 11]), and re-centrifuged at 100,000×*g* for 60 min. Pellet was resuspended with TET buffer (10 mM Tris-Cl, 0.1 M EDTA and 0.1 %Triton X-100 [pH 8.0]), clarified by low speed centrifugation (5000×*g* for 10 min) and then centrifuged at 100,000×*g* for 60 min. The new pellet was resuspended with 5 mL of acidic solution (50 mM glycine, 0.1% Triton X-100 [pH 2.5]), incubated for 30 min at room temperature and centrifuged at 100,000×*g*, 60 min. Pellets were washed twice with TE buffer (10 mM Tris-Cl and 0.1 M EDTA [pH 8.0] and finally centrifuged at 100,000×*g* for 60 min to recover intact HBB complexes.

### Electron microscopy and immunogold labeling

Flagellar HBB complexes were analyzed with immunogold electron microscopy for presence of entrapped MakA-Top7-mGFP chimeras within the flagellar channel. Purified HBB were seeded on carbon coated Formvar grids and samples were treated with rabbit anti-MakA polyclonal serum (1:100) and mouse anti-GFP antibodies (1:100). After 30 min. incubation at room temperature, samples were washed with TE buffer (pH 8.0) and probed with colloidal gold conjugated anti-rabbit (5 nm) and anti-mouse antibodies (10 nm) (SPI supplies). Samples were negatively stained with 1% Uranyl acetate and analyzed using a JEOL 2000EX electron microscope (JEOL Co., Ltd., Akishima).

### Data availability

The atomic coordinates and structure factors for MakA have been deposited in the Protein Data Bank under accession code 6EZV. DNA sequencing of *V. cholerae* mutants have been deposited in figshare https://figshare.com/s/813c33372e8a0169929b (2018).

## Electronic supplementary material

Supplementary Information
