# Supplementary material for: Flagella-mediated secretion of a novel Vibrio cholerae cytotoxin affecting both vertebrate and invertebrate hosts
Source: Commun Biol. 2018 Jun 7;1:59. doi: 10.1038/s42003-018-0065-z (PMC6123715; doi:10.1038/s42003-018-0065-z)
Supplement: Supplementary file 1 — Supplementary Information [file 42003_2018_65_MOESM1_ESM.pdf]

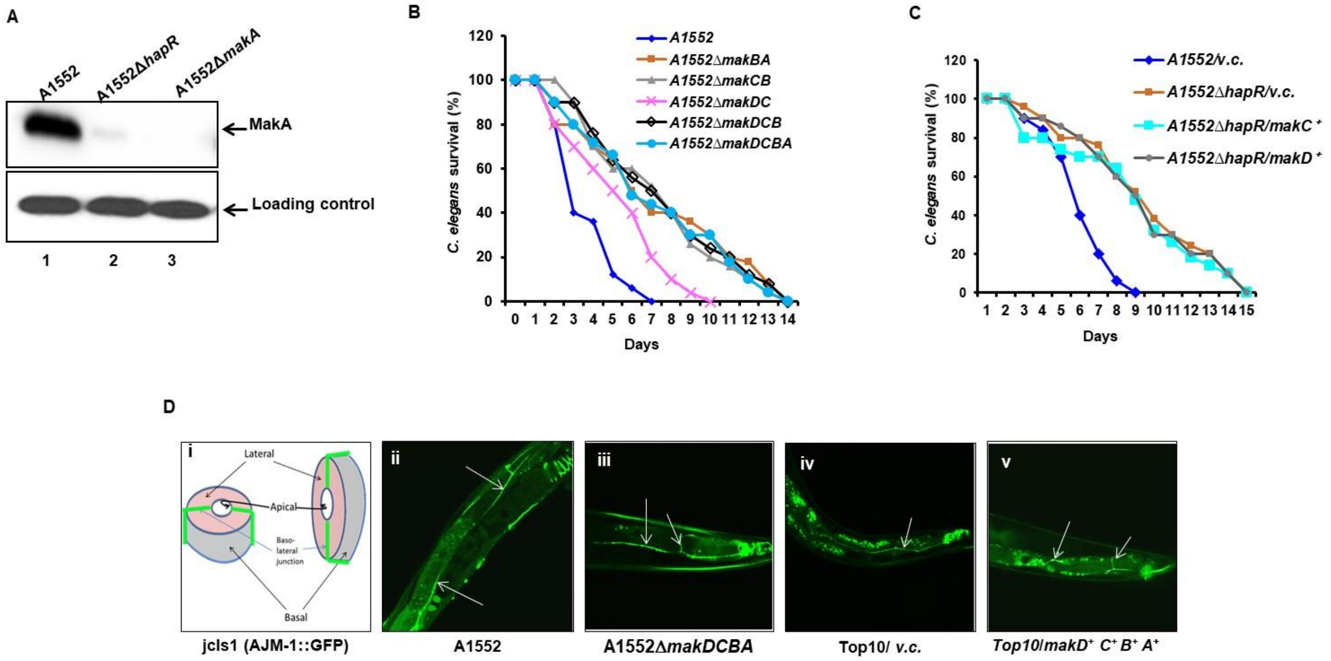

Supplementary Figure 1: Immunoblot analysis of MakA expression in the wild type A1552 strain and its quorum sensing regulator HapR mutant, *C. elegans* killing assay and visualization of *C. elegans* intestinal junction by fluorescent microscopic analysis. A) Immunoblot analysis of MakA expression using MakA polyclonal antiserum showing dramatically reduced level of MakA in the Δ*hapR* mutant (upper panel). Non-specific immunoblot reaction band detected was used as an internal loading control (lower panel). B) *C. elegans* killing by A1552 and its *mak* operon mutants; C) *C. elegans* survival upon feeding on the A1552Δ*hapR* mutant expressing *makC* or *makD* genes; D) Morphology of *C. elegans* intestine and localization of intestinal junctions. Schematic drawing showing the sites of *C. elegans* intestinal cell junctions (i), fluorescent photomicrographs (ii-v) of *C. elegans*. Arrows show the junctions of intestinal cells.

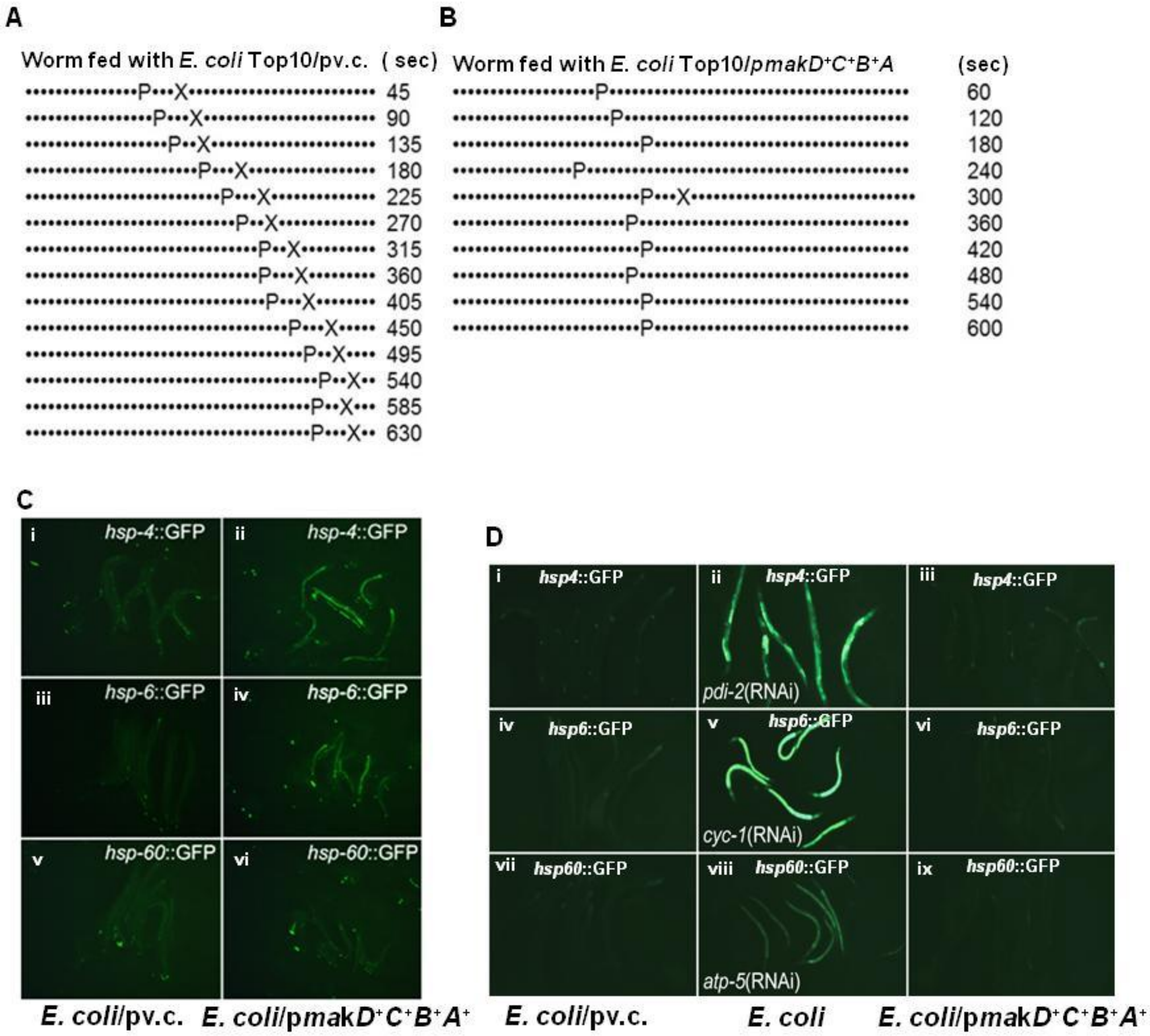

Supplementary Figure 2. Analyses of of consecutive defecation cycles and stress responses of *C. elegans* treated with either *E. coli* Top10/vector control or *E. coli* Top10/*makD*$^{+}$*C*$^{+}$*B*$^{+}$*A*$^{+}$. The dotted lines (each dot represents 1 sec) illustrate the continuous observation of an individual *C. elegans* and the occurrences in time of contraction of posterior body-wall muscles (P) or of expulsions (X) during defecation. A) Representative ethogram of *C. elegans* fed on *E. coli* Top10/vector control bacteria during a 630 sec observation. B) Representative ethogram of *C. elegans* fed with *E. coli* expressing the *mak*$^{+}$ operon during a 600 sec observation. The defecation cycles of *C. elegans* were representative of three independent experiments. C and D) Analysis of endoplasmic reticulum (ER) and mitochondrial stress responses in worms fed on bacteria with or without the *mak* operon. *hsp-4*::GFP is a marker for the ER unfolded protein response; *hsp-6*::GFP and *hsp-60*::GFP are both markers for the mitochondrial unfolded protein response. Micrographs of representative *C. elegans* worms viewed with fluorescence optics. C) Panels i, iii and v show worms fed on the non-pathogenic *E. coli* Top10/vector control strain. Panels ii, iv and vi show worms fed on the *E. coli* Top10/*makD*$^{+}$*C*$^{+}$*B*$^{+}$*A*$^{+}$ strain expressing all four Mak proteins. D) Panels i, ii, and iii worms harbouring an *hsp-4*::*gfp* transgene; panels iv, v, and vi show worms harbouring an *hsp-6*::*gfp* transgene; panels vii, viii, and ix show worms harbouring an *hsp-60*::*gfp* transgene. Panels i, iv, and vii show worms fed with the non-pathogenic *E. coli* Top10/vector control strain. Panels iii, vi, and ix show worms fed the *E. coli* Top10/*makD*$^{+}$*C*$^{+}$*B*$^{+}$*A*$^{+}$ strain expressing all four Mak proteins. Panels ii, v, and viii show worms subjected to RNAi of *pdi*-2, *cyc*-1 and *atp*-5 respectively.

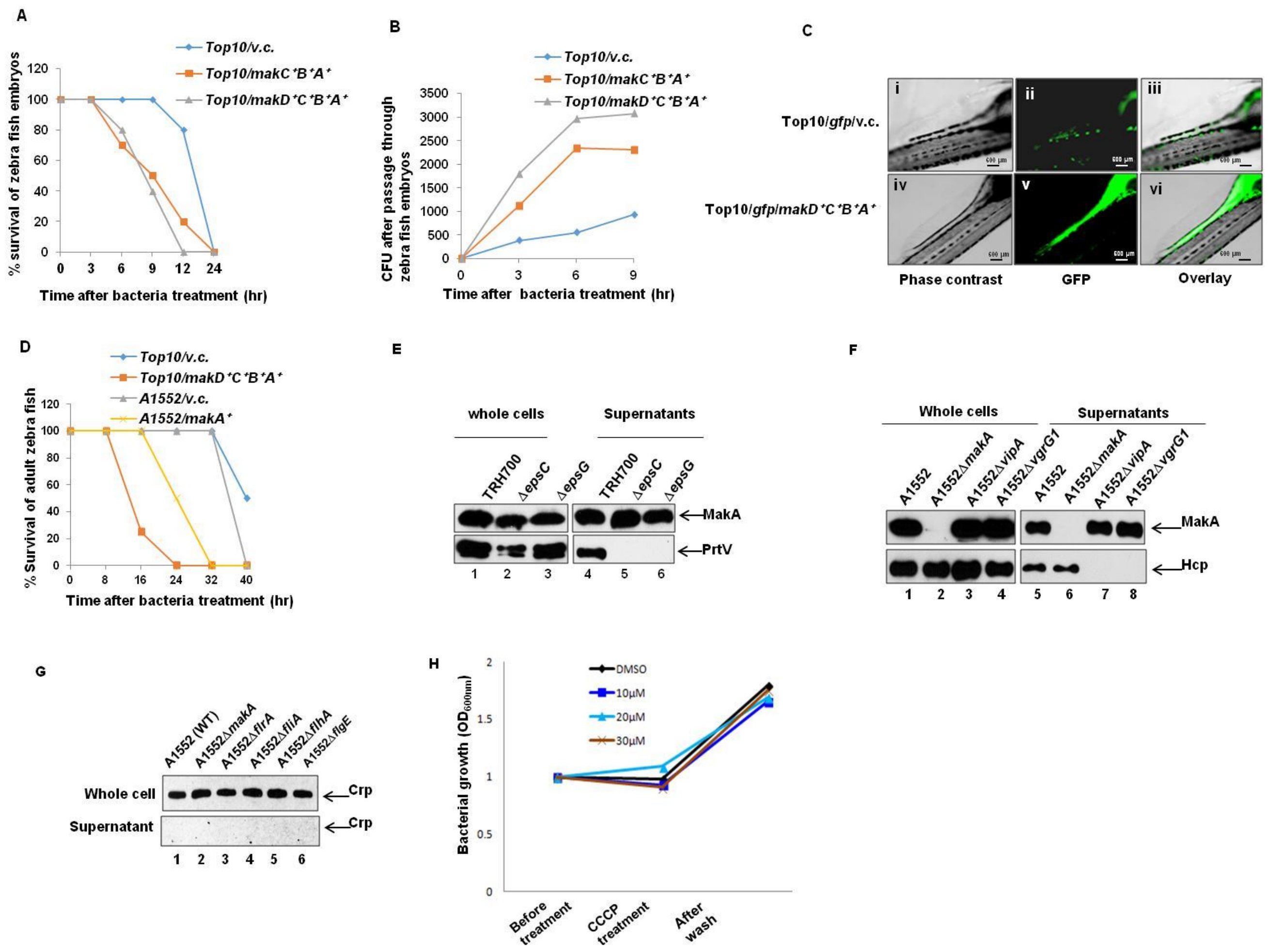

Supplementary Figure 3. Effect of MakA on zebrafish embryos and adult zebrafish and immunoblot analysis of MakA expression and secretion from different secretion system mutants of *V. cholerae* A) Survival of zebrafish embryos treated with 10^6 CFU/ml *E. coli* Top10 harboring either vector control or p*makC*$^+$*B*$^+$*A*$^+$ or p*makD*$^+$*C*$^+$*B*$^+$*A*$^+$ operon at different time points; B) Enumeration of bacteria internalized by zebrafish embryos at different time points; C) Fluorescence micrographs showing bacterial colonization of zebrafish embryo intestine after 9 h treatment: phase contrast (i and iv); fluorescence (ii and v), overlay (iii and vi); D) Survival of adult zebrafish treated with 10^7 CFU/ml *E. coli* Top10 or *V. cholerae* strain A1552 harbouring vector control or *pmakD*$^+$*C*$^+$*B*$^+$*A*$^+$ plasmid at different time points; E) Immunoblot analysis of MakA expression and secretion in the wild type *V. cholerae* O1 strain TRH700 and its T2SS mutant derivatives, Δ*epsC* and Δ*epsG* (upper panels). Secretion of PrtV as a T2SS substrate control (lower panel); F) Immunoblot analysis of MakA expression and secretion in the wild type *V. cholerae* O1 strain A1552 and its T6SS mutant derivatives Δ*vipA* and Δ*vgrG1* (upper panels). Secretion of Hcp as a T6SS substrate control (lower panel); G) Immunoblot analysis using anti-Crp antiserum in the culture supernatants of the wild type A1552 and its flagella cascade mutants as a marker for protein release due to cell lysis. H) Growth of bacteria before, during, and after treatment with indicated concentrations of CCCP.

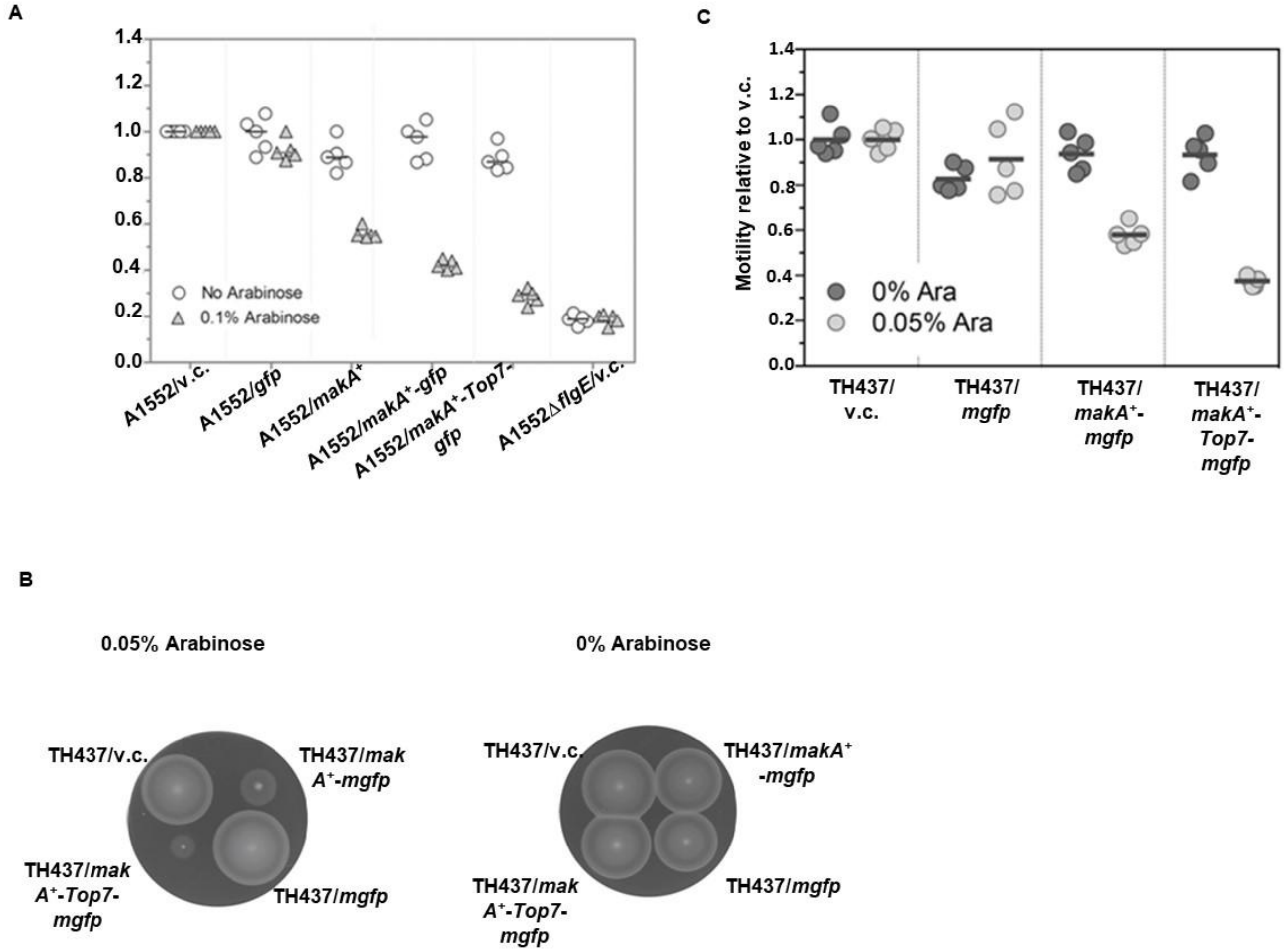

Supplementary Figure 4. Motility assay with *V. cholerae* and *S*. Typhimurium strains expressing MakA chimeric proteins. A) Quantitative analysis of motility by *V. cholerae* strains harboring different MakA fusion constructs. Diameters of bacterial zones were measured and plotted using GraphPad Prism. B) Motility assay with the wild type *S*. Typhimurium strain TH437 harboring plasmids p*makA*$^{+}$*-mgfp*, or p*makA*$^{+}$*-Top7-mgfp* using LA plates with or without 0.1% arabinose. C) Quantitative analysis of motility by *S. Typhimurium* strains harboring different MakA fusion constructs. Diameters of bacterial zones due to motility as shown in B) were measured and plotted using GraphPad Prism.

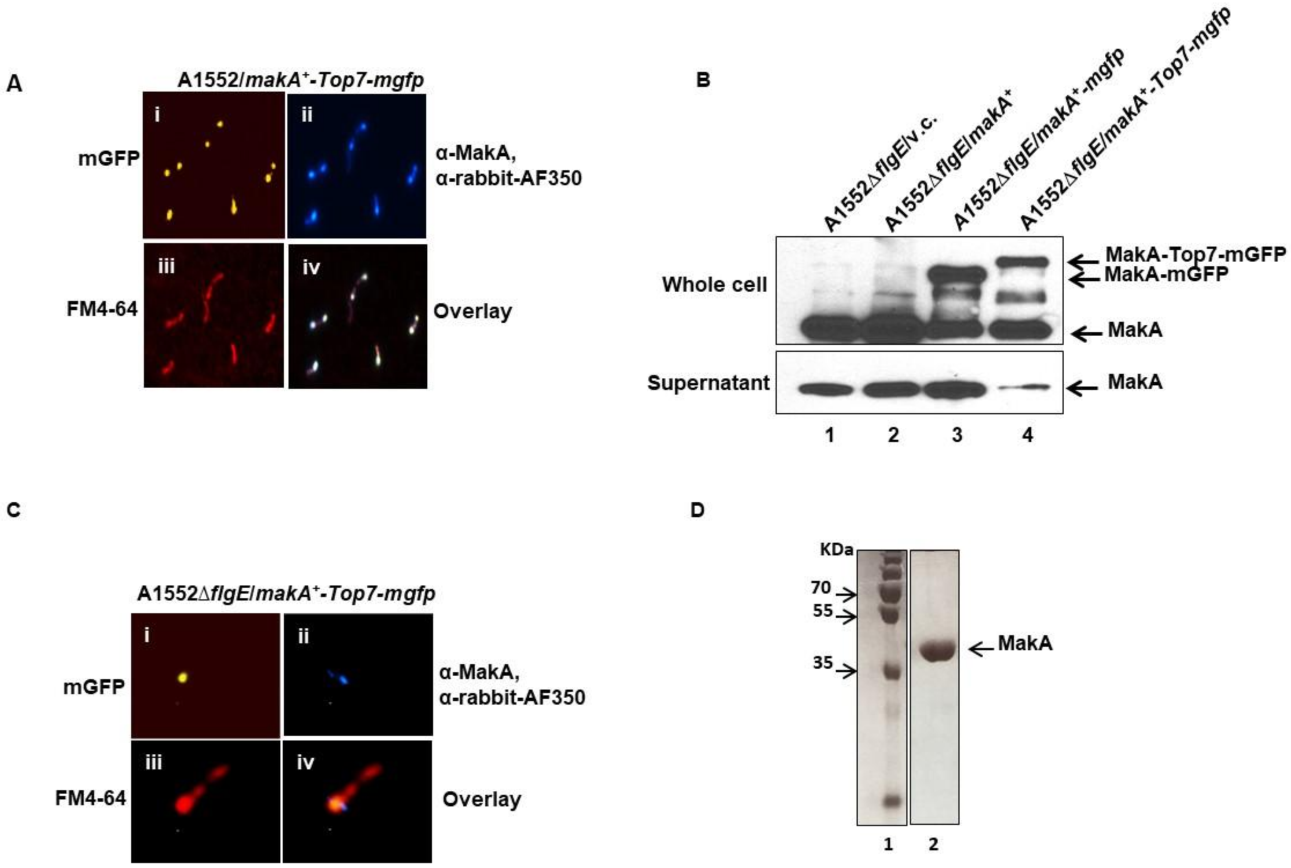

Supplementary Figure 5. Localization of MakA-Top7-mGFP chimeric protein in the wild type and the Δ*flgE* mutant of *V. cholerae*, analysis of secretion and expression levels. A) Images of A1552/p*makA*$^+$-*Top7-mgfp*: Polar localization of green fluorescence (i); Immunofluorescence with α-MakA/α-rabbit-AF350 antisera (ii); FM4-64 stained bacterial cells (iii); overlayed images (iv); B) Immunoblot analysis of MakA whole cell levels (upper panel) and the secreted levels (lower panel) in case of *V. cholerae* A1552Δ*flgE* harboring the *makA*$^+$ gene or chimeric *makA*$^+$ constructs with *mgfp* and/or *Top7*; C) Immunofluorescence microscopy images of the Δ*flgE* mutant harboring plasmid p*makA*$^+$-*Top7-mgfp* showing: Polar localization of mGFP fluorescence (i); Immunofluorescence assay with anti-MakA/anti-rabbit-AF350 antisera detecting polar localization of cellular MakA (ii); FM4-64 stained bacterial cells (iii); Overlay image (iv). D). SDS-PAGE Coomassie blue staining of purified MakA. Lane 1, molecular weight marker; lane 2, purified MakA.

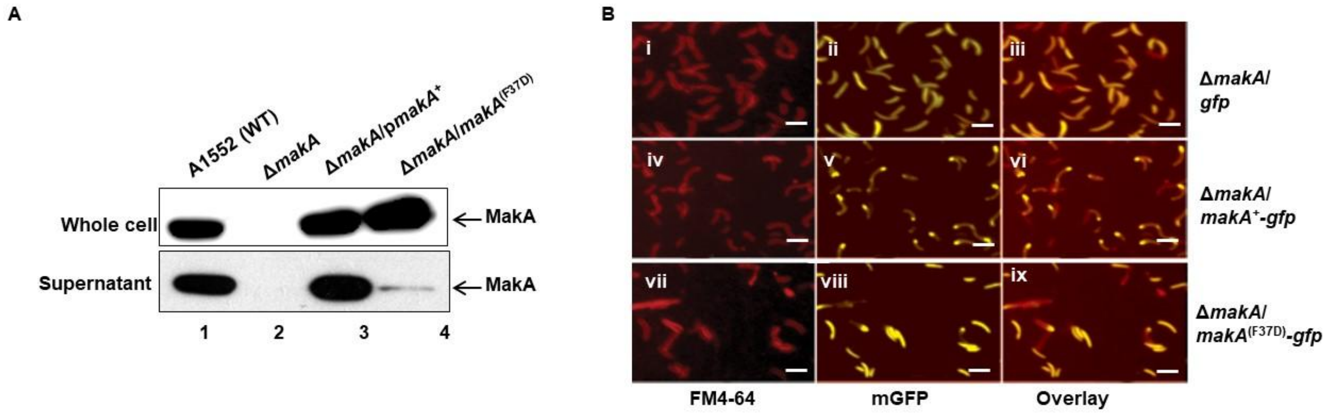

Supplementary Figure 6. Effect of F37D amino acid substitution on the secretion and polar localization of MakA and a crystal structure comparison of MakA with related structures A) immunoblot analysis of MakA and MakA$^{(F37D)}$ in *V. cholerae* A1552Δ*makA*; B) Fluorescence microscopy showing subcellular localization of mGFP (ii and iii); MakA-mGFP (v and vi); MakA$^{(F37D)}$-mGFP (viii and ix); FM4-64 staining for cell margins (i, iv, vii).

Supplementary Table 1. Bacterial strains and plasmids used in this study

Strain/ plasmid	Description/relevant characteristics	Source
Vibrio cholerae		
Vibrio cholerae A1552: serogroup O1, Rif^r		[1]
SNW2358	A1552Δ*hapR*	[2]
V5	*V. cholerae* non-O1 non-O139 (NOVC) serogroup O4, Sm^r	Swedish Institute of Infectious Diseases, Sweden (2004)
BL373	V:5Δ*hapR*	SNW lab collection
C6706	*Vibrio cholerae* serogroup O1, Rif^r	[3]
TAKA86	A1552Δ*flrA*	This study
TAKA11	A1552Δ*fliA*	This study
MDS012	A1552Δ*prtV*	This study
MDS002	A1552Δ*makD*	This study
MDS003	A1552Δ*makC*	This study
MDS004	A1552Δ*makB*	This study
MDS005	A1552Δ*makA*	This study
MDS006	A1552Δ*makDC*	This study
MDS007	A1552Δ*makCB*	This study
MDS008	A1552Δ*makDCB*	This study
MDS009	A1552Δ*makBA*	This study
MDS010	A1552Δ*makDCBA*	This study
MDS011	A1552Δ*flgE*	This study
MDS013	A1552Δ*flhA*	This study
TAKA17	A1552Δ*vgrG3*	[4]
SNW2644	A1552Δ*vipA*	[4]
TRH700	El Tor O1 biotype; wild type for T2SS	[5]
TRHΔ*epsC*	*epsC* deletion in TRH7000, Kan^r	[5]
TRHΔ*epsG*	*epsG* deletion in TRH7000, Kan^r	[5]

MDS110	A1552/pBAD18; Amp^r	This study
MDS111	A1552Δ*hapR*/pBAD18; Amp^r	This study
MDS112	A1552Δ*hapR*/pMDC001	This study
MDS113	A1552Δ*hapR*/pMDC002	This study
MDS114	A1552Δ*hapR*/pMDC003	This study
MDS115	A1552Δ*hapR*/pMDC004	This study
MDS116	A1552/pMDC004	This study
MDS117	A1552Δ*makA*/pMDC004	This study
MDS118	A1552Δ*makA*/pMDC008	This study
MDS119	A1552Δ*flgE/*pMDC004	This study
MDS120	A1552Δ*makA*/pMDC003	This study
MDS121	A1552Δ*makB*/pMDC004	This study
MDS122	A1552Δ*flgE/*pBAD18	This Study
MDS123	A1552Δ*makA*/pBad18	This Study
MDS210	A1552/pBSB268	This study
MDS211	A1552/pBSB454	This study
MDS214	A1552/pMDB113	This study
MDS215	A1552Δ*flgE/*pBSB268	This study
MDS212	A1552Δ*flgE*/pBSB454	This study
MDS213	A1552Δ*flgE*/pMDB113	This study
E. coli		
Top10, MDS610	F^- *mcrA* Δ*(mrr-hsdRMS-mcrBC) ϕ80lacZ*Δ*M15* Δ*lacX74 nupG recA1 araD139* Δ(*ara-leu*)*7697 galE15 galK16 rpsL*(*StrR*) *endA1* λ^-	Invitrogen inc.
SM10λpir	*thi thr leu tonA lacY supE recA*::RP4-2 Tc::Mu Km λpir	[6]
MDS611	Top10/pBAD18; Ap^r	This study
MDS612	Top10/pMDC001	This study
MDS613	Top10/pMDC002	This study
MDS614	Top10/pMDC003	This study

MDS615	Top10/pMDC004	This study
MDS616	Top10/pMDC005	This study
MDS617	Top10/pMDC006	This study
MDS618	Top10/pMDC007	This study
MDS619	Top10/pBAD18 +pDHS18	This study
MDS620	Top10/pMDC007+pDHS18	This study
Salmonella enterica **serovar Typhimurium**		
TH437	Wild type strain LT2	John Roth
TH3708	DEL1103(*tct-zfg*-3516::Tn*10*dTc-*fljB*5001::Mu*d*J) DEL1131(*fliA*5059::Tn*10*dTc-*fliO*5097::Mu*d*J) DEL1133(*flhAB*5056::Tn*10*dTc-*flhC*5213::Mu*d*J)(Δ*flhD*) DEL1137(*flgN*5220::Mu*d*J-*flgL*566::Tn*10*)	Kelly Hughes
EM77	Δ*invH-sprB*::FRT (Δ*spi-1*) Δ*sseA-ssaU*::FRT (Δ*spi-2*)	Kelly Hughes
TH17121	Δ*sseA-ssaV*::FRT Δ*invH-sprB*::FRT Δ*araBAD*1014::*flgE-bla* DEL1103[*tct-fljB*] DEL1131[*fliA-fliR*] DEL1133[*flhB-flhD*] DEL1137[*flgN-flgL*]	Kelly Hughes
MDS710	TH437/pBAD18; Vector control, Ap^r	This Study
MDS711	TH437/pMDC004	This Study
MDS712	TH3708/pMDC004	This Study
MDS713	EM77/pMDC004	This Study
MDS714	TH17121/pMDC004	This Study
Yersinia pseudotuberculosis		
YPIII(pIB102)	*Y. pseudotuberculosis* serotype III, wild type	[7]
YPIII[p-]	*Y. pseudotuberculosis* plasmid cured (T3SS mutant)	[7]
MDS811	YPIII(pIB102)/pMDC004	This study
MDS812	YPIII[p-]/pMDC004	This study
YPIII(pIB102) Δ*flhD*/pIBX	YPIII(pIB102) *flhD* deletion mutant	[8]
Plasmids		
pBAD18	Arabinose-inducible cloning vector; Ap^r	[9]

pCVD442	Suicide plasmid; R6K ori, mobRP4, bla, *sacB*; Ap^r	[10]
pDHS18	Constitutive GFP expression plasmid; Cm^r	SNW lab collection
pMDC001	*makD*::pBAD18	This Study
pMDC002	*makC*::pBAD18	This Study
pMDC003	*makB*::pBAD18	This Study
pMDC004	*makA*::pBAD18	This Study
pMDC005	*makBA::*pBAD18	This Study
pMDC006	*makCBA::*pBAD18	This Study
pMDC007	*makDCBA::*pBAD18	This Study
pMDC008	*makA*^{F37D}::pBAD18	This Study
pSEVA21	Ap^r	Esteban Martínez
pBSB268	P_{BAD}-mGFP::pSEVA121	This Study
pBS454	P_{BAD}-*makA*-m*gfp*::pSEVA121	This Study
pMDB113	P_{BAD}-*makA*-*Top7*-m*gfp*::pSEVA121	This Study
pMDD109	Δ*prtv*::pCVD442	This Study
pMDD110	Δ*makD*::pCVD442	This Study
pMDD111	Δ*makC*::pCVD442	This Study
pMDD112	Δ*makB*::pCVD442	This Study
pMDD113	Δ*makA*::pCVD442	This Study
pMDD114	Δ*makDC*::pCVD442	This Study
pMDD115	Δ*makCB*::pCVD442	This Study
pMDD116	Δ*makDCB*::pCVD442	This Study
pMDD117	Δ*makBA*::pCVD442	This Study
pMDD118	Δ*makDCBA*::pCVD442	This Study
pMDD119	Δ*flgE*::pCVD442	This Study
pMDD120	Δ*flhA*::pCVD442	This Study

Supplementary Table 2. Primers used in this study

Name	Sequence 5′→ 3′	Restriction site	For construction of
OMD098	CATTCTAGAACATAGATATCATTTATCCATGTCG	*XbaI*	*ΔprtV*
OMD099	CCCATCCACTATAAACTAACATATGGCGGCAGCTAATAGCG	*XbaI*	*ΔprtV*
OMD100	TGTTAGTTTATAGTGGATGGGCTGTAATTCTTCCTTCTCCTTCC		*ΔprtV*
OMD101	CTATCTAGACTGTTTGCAGAGGAGAATCGGCTG	*XbaI*	*ΔprtV*
O2066A	CGCTCTAGAGAAATCATCCTGAATCAGCCT	*XbaI*	Δ*fliA*
O2066B	CCCATCCACTATAAACTAACACAATCTATGCAGCGGCTAAGA		Δ*fliA*
O2066C	TGTTAGTTTATAGTGGATGGGCTATTGATCGTATGTAAGCGC		Δ*fliA*
O2066D	CGCTCTAGATGGGACTGATTCGTGCATTTG	*XbaI*	Δ*fliA*
O2137A	CGCTGTAGAGATATCATCATTAGCCTAC	*XbaI*	Δ*flrA*
O2137B	CCCATCCACTATAAACTAACACGCTAAACTCTGCATAGGT		Δ*flrA*
O2137C	TGTTAGTTTATAGTGGATGGGGAAGAGCCGCAGGAGTTTG		Δ*flrA*
O2137D	CGCTCTAGACTTCTGGGTTGGCTTCACG	*XbaI*	Δ*flrA*
OMD118	5’AATTCTAGATGATGGCATTGGCAAGATGAAC	*XbaI*	*ΔflgE*
OMD119	5’CCCATCCACTATAAACTAACAGAGTAATTCTCCTATCTAGCT		*ΔflgE*
OMD120	5’TGTTAGTTTATAGTGGATGGGCTGCAGATTCGTTAATCCTCTTC		*ΔflgE*
OMD121	5’AATTCTAGATCCCGTTTCATCGATTCGTAAG FLGE D	*XbaI*	*ΔflgE*
OMD122	5’AATTCTAGATTGAGGTTGAACAGCTCGAAT	*XbaI*	*ΔflhA*
OMD123	5’CCCATCCACTATAAACTAACAAAATTTCTGGCTGTCACTGAAGG		*ΔflhA*
OMD124	5’TGTTAGTTTATAGTGGATGGGTCGTCCGCACGTTTTTTAAAG		*ΔflhA*
OMD125	5’AATTCTAGAGGAGAGTCAGGACGTTGACGC	*XbaI*	*ΔflhA*
OMD102	5´CGCTCTAGAGAACTAGCAGCAGTTATACTT	*XbaI*	*ΔmakD*
OMD103	5´CCCATCCACTATAAACTAACACATCCAATACCTACTGACGTT		*ΔmakD*
OMD104	5´TGTTAGTTTATAGTGGATGGGCTCCATCTAAATAAGGAACATTGTATG		*ΔmakD*
OMD105	5´CGCTCTAGACGGTCAGCATATTTAATGCGG	*XbaI*	*ΔmakD*
OMD106	5´CGCTCTAGAGGTAAAACCCAAAAACCATTCGAA	*XbaI*	*ΔmakC*
OMD107	5´CCCATCCACTATAAACTAACATGCTGCATCTACGACCATGAG		*ΔmakC*
OMD108	5´TGTTAGTTTATAGTGGATGGGGCGAACTCATGTTAAGCC		*ΔmakC*
OMD109	5´CGCTCTAGACTGTTGCAATGCGTACCATTG	*XbaI*	*ΔmakC*
OMD110	5´CGCTCTAGATCTCGAAGTTCAATAACTCCATCT	*XbaI*	*ΔmakB*

OMD111	5´CCCATCCACTATAAACTAACACATGAGTTCGCCCTCTACAGT		*ΔmakB*
OMD112	5´TGTTAGTTTATAGTGGATGGGAGGAGATACACAATGTCACAACAA		*ΔmakB*
OMD113	5´CGCTCTAGACGCACCATTGACTAAGTCATC	*XbaI*	*ΔmakB*
OMD114	5´ATCTCTAGATGGTCACTATTTCCTACGGTGTCG	*XbaI*	*ΔmakA*
OMD115	5´CCCATCCACTATAAACTAACACAGATTGAGTGGTTTGCTGTGTTGGG		*ΔmakA*
OMD116	5´TGTTAGTTTATAGTGGATGGGCGAAAGAGTTAGCACCTGCAG		*ΔmakA*
OMD117	5´CGCTCTAGATGGCGATAATTTCTGCTTGGATTG	*XbaI*	*ΔmakA*
OMC100	5’-AAAGAGCTCATCAGTACAGTGATACCCTGTG	*SacI*	*makD*$^{+}$
OMC101	5’-GTCTCTAGATTATTGAACTTCGAGATAAGGTG	*XbaI*	*makD*$^{+}$
OMC102	5’-ATAGAGCTCCAATAACTCCATCTAAATAAGG	*SacI*	*makC*$^{+}$
OMC103	5’-GTCTCTAGACTACAGTGAAATAACGAGGAAAG	*XbaI*	*makC*$^{+}$
OMC104	5’-ATAGAGCTCCCTCGTTATTTCACTGTAGAG	*SacI*	*maKB*$^{+}$
OMC105	5’-ATATCTAGACTCCTTTACTGTTCTTGAATGGTG	*XbaI*	*makB*$^{+}$
OMC106	5’-ATAGAGCTCACCATTCAAGAACAGTAAAGG	*SacI*	*makA*$^{+}$
OMC107	5’-ATATCTAGATTAAGCTGCTTGTTTTACTGC	*XbaI*	*makA*$^{+}$
OMC108	5'-AGTGCCATGCCATTTTAAACACACAAGATACGCCACCTACGG		*makA*$^{F37D+}$
OMC109	5'-CCGTAGGTGGCGTATCTTGTGTGTTTAAAATGGCATGGCACT		*makA*$^{F37D+}$
BSBP237	AAGGAGATATACCCATGGTGATGAAAGGAGAAGAACTTTTCACTGGA		*gfp*$^{+}$ - BSB268
BSBP142	CCGGGTACCGAGCTCTTATTTGATGCCTCTAGATTTAAATGCT		*gfp*$^{+}$ -pBSB268
BSBP275	AACCATTACCTGTCGACACAATCTAAACTTTCGAAAGATCCCAACGAAAAG		*mgfp*$^{+}$
BSBP276	CTTTTCGTTGGGATCTTTCGAAAGTTTAGATTGTGTCGACAGGTAATGGTT		*mgfp*$^{+}$
BSBP283	CACCATGGGTATATCTCCTTCTTA		pBSB454
BSBP284	GCTAGCAAAGGAGAAGAACTTTTCA		pBSB454
OMC110	AGATATACCCATGGTGATGTCACAACAAGTTACTCA		*makA*$^{+}$*-gfp*
OMC111	TTCTCCTTTGCTAGCAGCTGCTTGTTTTACTG		*makA*$^{+}$*-gfp*
OMC112	GCTAGCAAAGGAGAAGAAC		pMDB113
OMC113	AGCTGCTTGTTTTACTGC		pMDB113
OMC114	GCAGTAAAACAAGCAGCTATGGATATCCAGGTTCAAGTG		*Top7 insertion*
OMC115	GTTCTTCTCCTTTGCTAGCCTCTAACTGTCCTTCCACTGTC		*Top7 insertion*
KPC001	GCTTTCCATGGGTTCACAACAAGTTACTCAGTTGAACC	*NcoI*	*makA*$^{+}$*-his*
KPC002	GCTTTGGTACCTTAAGCTGCTTGTTTTACTGCAGG	*Acc65I*	*makA*$^{+}$*-his*

Supplementary Table 3. **Data collection and refinement statistics**

	MakA SeMet	MakA native
**Data collection**		
Wavelength	0.9718	0.9677
Space group	$P2_1$	$P2_1$
Cell dimensions		
a, *b*, *c* (Å)	64.56, 36.81 73.50	64.57, 36.53, 73.55
β (°)	91.59	91.25
Molecules in asymmetric unit	1	1
Solvent content (%)	45.1	44.7
Resolution (Å)	47.83-2.00	48.0-1.90
Highest resolution shell	2.05-2.00	1.94-1.90
R_{merge}*	0.125 (0.788)	0.079 (0.785)
*Rpim**	0.050 (0.312)	0.047 (0.46)
$I / \sigma I$ *	12.0 (3.9)	12.5 (2.5)
*CC1/2**	0.997 (0.935)	0.987 (0.850)
Completeness (%)*	98.3 (97.3)	94.5 (95.9)
Redundancy*	13.8 (14.2)	7.4 (7.6)
**Refinement**		
Resolution (Å)		48.0-1.90
No. reflections (work/test)		25871 /1290
R_{work} / R_{free}		0.195/0.249
No. atoms		
Protein		2637
Ligands (glycerol, acatete, cacodylate)		25
Water		134
B-factors (Å^2)		
Protein		36.1
Ligands		51.3
Water		34.8
R.m.s. deviations		
Bond lengths (Å)		0.011
Bond angles (°)		1.024
Ramachandran plot (%)		
Favoured, allowed		99.0, 1.0
PDB code		6EZV

*Values in parentheses are for the highest-resolution shell.

Supplementary References:

1 Yildiz, F. H. & Schoolnik, G. K. Role of rpoS in stress survival and virulence of *Vibrio cholerae. J Bacteriol* **180**, 773-784 (1998).

2 Ishikawa, T., Rompikuntal, P. K., Lindmark, B., Milton, D. L. & Wai, S. N. Quorum sensing regulation of the two hcp alleles in *Vibrio cholerae* O1 strains. *PLoS One* **4**, e6734, doi:10.1371/journal.pone.0006734 (2009).

3 Thelin, K. H. & Taylor, R. K. Toxin-coregulated pilus, but not mannose-sensitive hemagglutinin, is required for colonization by *Vibrio cholerae* O1 El Tor biotype and O139 strains. *Infection and immunity* **64**, 2853-2856 (1996).

4 Ishikawa, T. *et al.* Pathoadaptive conditional regulation of the type VI secretion system in *Vibrio cholerae* O1 strains. *Infection and immunity* **80**, 575-584, doi:10.1128/IAI.05510-11 (2012).

5 Johnson, T. L. *et al.* The Type II secretion system delivers matrix proteins for biofilm formation by *Vibrio cholerae*. *J Bacteriol* **196**, 4245-4252, doi:10.1128/JB.01944-14 (2014).

6 Miller, V. L., Taylor, R. K. & Mekalanos, J. J. Cholera toxin transcriptional activator toxR is a transmembrane DNA binding protein. *Cell* **48**, 271-279 (1987).

7 Bolin, I. & Wolf-Watz, H. Molecular cloning of the temperature-inducible outer membrane protein 1 of *Yersinia pseudotuberculosis. Infection and immunity* **43**, 72-78 (1984).

8 Avican, K. *et al.* Reprogramming of Yersinia from virulent to persistent mode revealed by complex in vivo RNA-seq analysis. *PLoS Pathog* **11**, e1004600, doi:10.1371/journal.ppat.1004600 (2015).

9 Guzman, L. M., Belin, D., Carson, M. J. & Beckwith, J. Tight regulation, modulation, and high-level expression by vectors containing the arabinose PBAD promoter. *J Bacteriol* **177**, 4121-4130 (1995).

10 Donnenberg, M. S. & Kaper, J. B. Construction of an eae deletion mutant of enteropathogenic *Escherichia coli* by using a positive-selection suicide vector. *Infection and immunity* **59**, 4310-4317 (1991).
